# Carotid Intima-Media Thickness in Healthy Children and Adolescents: Normative Data and Systematic Literature Review

**DOI:** 10.3389/fcvm.2020.597768

**Published:** 2020-11-26

**Authors:** Ana Drole Torkar, Emil Plesnik, Urh Groselj, Tadej Battelino, Primoz Kotnik

**Affiliations:** ^1^University Children's Hospital, Department of Endocrinology, Diabetes and Metabolism, Ljubljana University Medical Centre, Ljubljana, Slovenia; ^2^Better d.o.o., Ljubljana, Slovenia; ^3^Faculty of Medicine, University of Ljubljana, Ljubljana, Slovenia

**Keywords:** RF-QIMT, cIMT, intima-media thickness, atherosclerosis, vascular health, primary prevention

## Abstract

**Objectives:** Early identification of children at risk of atherosclerosis is of paramount importance for implementing primary preventive measures addressing vascular health. Carotid intima-media thickness (cIMT) is a non-invasive biomarker of atherosclerosis. Semiautomatic radiofrequency-based software-guided technique quality intima-media thickness (RF-QIMT) was used to determine cIMT normative values in a healthy cohort of Caucasian children aged 6 to 18 years.

**Study design:** In a cross-sectional study, data on age, chronic illness, medication use, and pubertal status was acquired by a questioner. Anthropometric and blood pressure measurements were performed by standardized methods and trained medical personnel. cIMT of the right common carotid artery far wall (1 centimeter proximal to bifurcation) was determined using a multifrequency (3–13 MHz) electronic linear array transducer SL1543, a portable ultrasound device (MyLab Gamma Esaote, Genoa, Italy), and RF-QIMT software. A systematic review of the published normal cIMT in children was done using PRISMA methodology, and identified normative values were compared to those obtained in the presented study.

**Results:** 1137 non-obese normotensive children (males: *n* = 512; mean age 12.04 ± 3.52 years, females: *n* = 625, mean age 12.98 ± 3.83 years) were included. Gender-, age-, and height-specific mean cIMT percentile tables, percentile charts, and LMS tables for the RF-QIMT method were provided. They were comparable to the previously published data on mean cIMT gained by other validated ultrasound imaging techniques. cIMT increased with age, height, hip circumference, and BMI and was higher in males.

**Conclusions:** Gender-, age-, and height-specific normative cIMT values, using the semiautomatic software-guided RF-QIMT technique, in children aged 6 to 18 years were developed and validated in respect to the previously published pediatric normative cIMT data. It is suggested that the investigated method could be used for the estimation of atherosclerotic risk in children, especially in epidemiological studies.

## Introduction

The prodromal stages of atherosclerotic (AS) lesions are present early in life (1–3) and precede clinically manifested cardiovascular disease (CVD) (4, 5). The process progresses into adulthood when fatty streaks and fibrous lesions can occupy 25–40% of the aortic intima in the third decade of life even in presumably healthy subjects (6).

Ultrasonographic (US) evaluation of subclinical vascular disease can be useful for refining CVD risk assessment (7). US measurement of carotid intima-media thickness (cIMT) is a non-invasive risk stratification tool as it represents a surrogate biomarker of generalized AS throughout the arterial tree (8, 9). It can be used to quantify the extent of subclinical AS changes in the vascular wall early in its development and to monitor change over time (10, 11).

Data on physiological cIMT increase in the pediatric population is, to some degree, conflicting. In childhood, mild degrees of intima-media thickening reflect a compensatory adaptation of intimal and medial layers to pressure and flow in the absence of AS lesions (12–15). Therefore, cIMT physiologically increases in children in association with age and growth (5, 7, 15–19), male gender (5, 7, 19, 20), pubertal maturation (20), as well as ethnicity (7, 21), and geographical factors (22, 23).

Nevertheless, cIMT increase beyond a certain level represents vascular remodeling in response to known AS risk factors (13, 18, 24–26). Childhood- and adulthood-onset risk factors contribute to increased cIMT in later life (27). Importantly, cIMT can be partially reduced by influencing those risk factors (28, 29), which further supports the efforts to address modifiable cardiovascular risk factors early in life (4).

American Heart Association recommendations for non-invasive assessment of AS in children suggested using cIMT for the risk estimation in the pediatric population a decade ago (4). However, high-quality age and gender-specific data on the normal values for cIMT in the pediatric population were needed (30). Normative values depend on the US cIMT measurement technique, its technological challenges (18, 19), and the site of the measurement (12, 21). The available methods for measuring cIMT are highly variable in terms of signal processing and image analysis. cIMT can be measured trough B-mode ultrasonography, where cIMT is calculated from a distance between echogenic lines of the arterial wall that present lumen-intima and media-adventitia borders; it can be measured manually using electronic calipers with a visually identified single point of the arterial wall. Semi-automated edge detection programs have been suggested as a better approach to achieve the accuracy of the cIMT measurement as the examiner is enabled to redirect the algorithm in cases of improper edge detection (31). Edge-tracking software use algorithms in image analysis to automatically identify the borders at multiple points along the arterial wall. There are several manual or automated off-line image interpretation tools and, more recently, also real-time cIMT interpretation software programs available (4, 5, 7, 24, 31). Automated edge detection based on radiofrequency signal tracking is one of the most accurate methods (24). It is also shown that semi-automated radiofrequency-based cIMT measurements performed by non-radiology and non-cardiology specialists could be a reliable, fast, feasible, and easy-to-learn method when appropriately quality-controlled as it is less dependant on the B-mode image quality and on the experience in vascular US of the examiner (4, 32, 33).

We aimed to measure cIMT using a semi-automatic radiofrequency-based, software-guided technique, quality intima-media thickness (RF-QIMT), in a healthy cohort of Caucasian children and adolescents aged 6 to 18 years. Gender-, age-, and height-specific reference normative values were developed. The influence of pubertal status, obesity, and blood pressure on cIMT was analyzed. Additionally, we systematically compared our data to previously published data on cIMT obtained by noninvasive US methods.

## Methods

### Subjects and Study Design

The National Medical Ethics Committee fully approved the study (No. of the approval: 0120-357/2017-/9). Healthy volunteers were included after the informing process and written parental consent. A questionnaire acquired information on age, chronic illness, and medication use, and pubertal development status was reported using self- and parental-assessment based on provided Tanner image scale (34).

Altogether, we examined 1,241 subjects aged from 6 to 18 years for the cIMT measurement. We excluded 39 subjects from further analysis due to chronic illness (*N* = 30: asthma (*N* = 10), diabetes type I (*N* = 2), autoimmune thyroiditis (*N* = 3), self-reported precocious puberty (*N* = 4), pre-diagnosed familial hypercholesterolemia (FH) through national FH screening program (35) (*N* = 5), and other chronic illness (*N* = 6) and due to poor participation and low quality of image acquisition (*N* = 9). Data collection took place from October 2017 to April 2019. Trained medical personnel, using validated equipment, took anthropometric measurements (weight, height, waist, and hip circumferences) rounded to the first decimal place, and indexes were calculated accordingly (BMI, waist circumference to height ratio, waist to hip circumference ratio). We performed blood pressure measurements using a standardized device and cuffs after the volunteer was resting for at least 5 min. Blood pressure was measured three times, with 1-min rest intervals; reported blood pressure was an average of the three obtained values.

### cIMT Measurement

cIMT measurement was done using ultrasound device MyLab Gamma Esaote, Genoa, Italy, equipped with a multifrequency (3–13 MHz) electronic linear array transducer SL1543 and utilizing a radiofrequency-based ultrasound system (RF-quality intima-media thickness, RF-QIMT, Esaote, Genoa, Italy) (33), for the real-time measurement of the intima-media thickness of the right common carotid artery (CCA) far wall imaged in a longitudinal view from a lateral approach. A single-side cIMT measurement was performed to decrease the examination burden on children as it was previously proven sufficient to detect age- and gender-specific cIMT values in children (19). The measurement was performed according to manufacturer instructions with RF-QIMT software. A vertical reference line was placed on the bulb origin and the horizontal reference marker in the center of the artery lumen as presented in Supplementary Figure 1. The reported average cIMT was calculated from six consecutive measurements acquired in a 1.5-centimeter-long region of interest placed one centimeter proximal of the vertical reference line where the standard deviation between measurements did not exceed 20 μm. All measurements were documented and stored digitally. Three pediatric specialists performed measurements following the Mannheim protocol (36).

To calculate the intra- and interobserver variability of the measurements, a subgroup of 38 volunteers was assessed twice by observer one and twice by either observer two and/or observer *three*. The observers were blinded to each other's results.

*Measurement variability and within-observer agreement* were assessed using Bland–Altman plots.

### Statistical Analysis Protocol

We used R-project 3.6.0 software (https://cran.r-project.org) in all statistical calculations and analysis.

*Descriptive statistics* for age, gender, pubertal developmental stage, anthropometrics, obesity measures, pulse rate, blood pressure, and standard deviation score (SDS) values were calculated (37). Frequency distributions and prevalence of independent variables elevations were determined using different available reference standards published in the literature, and comparison was made.

**Figure 1:**
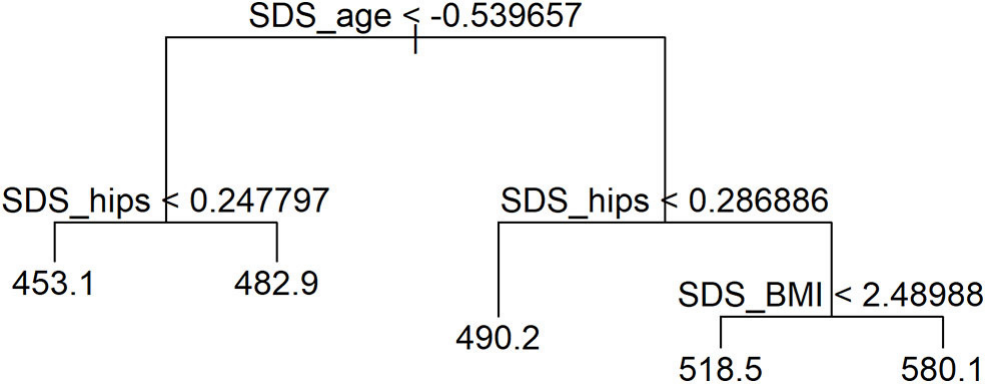
Regression tree model analyzing interactions of independent variables.

*Association of covariates* was assessed with a one-way ANOVA test. Statistical significance was set at *p* < 0.05. A multiple linear regression model was constructed to analyze the effects of independent variables on the cIMT using a backward elimination approach. The missing values were imputed by applying a non-parametric, mixed-type imputation method based on random forests (38). The multiple linear model was optimized, and interactions were researched. All independent variables with high multi-collinearity by the variance inflation factor (39) were discarded: SDS value of body weight, SDS value of waist circumference, and waist circumference to height ratio. To assess whether complex interactions were indicated between independent variables, a regression tree model was fitted as presented in Figure 1, and it indicated that the interaction structure of the data was not complex. Independent numeric variables were standardized using available references (40–42); age was standardized by subtracting the mean and dividing by SD. All variables were inspected for outliers outside of the ±3 SD of the sample median, and altogether 53 boys and girls were excluded during this process.

*Reference values of cIMT measured with the RF-QIMT method* were calculated for gender, age, and height. The values were tested for normal distribution by the Shapiro–Wilk test, and the LMS method (43) was used for normative values construction. Values of cIMT above +3 SD and below −3 SD of the sample median and subjects with BMI and blood pressure above + 2 SD (*N* = 65, 37 boys and 28 girls) were excluded from the construction of the normative value model (44). The LMS method was used for the description of pediatric anthropometric data as it allows for calculating percentiles and accurately normalized SDS values accounting for non-linearity and skewed distributions of the reference data set (5, 45). The reference LMS tables display the mean (M), the coefficient of variation (S), and the measure of skewness (L). The equation can calculate the SDS for each individual: *SDS* = [Y/M(t)] L(t)-1/ [L(t)^*^S(t)]; where *Y* is the individual measurement, and *L, M*, and *S* originate from the specific reference values for each age (*t*). The equation calculates the corresponding percentile value for each individual with a specific SDS: *p*th *percentile* = *M(t) [1*+*L(t) S(t)SDSp]1/L(t)*, where *SDSp* is the SDS value converted from the Gaussian table corresponding to the *p*th percentile. This formula converts standard Gaussian percentiles to LMS percentiles of the selected model.

### A Systematic Review of Published Literature on Normative cIMT Values in the Pediatric Population

PRISMA reporting methodology was used for the literature search performed on July 22, 2019, and updated on December 27, 2019. We searched for published cIMT normative data using keywords “carotid intima-media thickness” and “healthy children and adolescents” in the English language in PubMed, Mendeley, and Cochrane databases. The report is available in Supplementary Figure 2.

## Results

### Characteristics of the Participants

Altogether 1,202 healthy Caucasian children and adolescents aged 6 to 18 years (males: *n* = 549; mean age 12.04 ± 3.52 years, females: *n* = 653, mean age 12.98 ± 3.83 years) were included in the analysis. Detailed descriptive statistics of the study group are presented in Table 1, and pubertal developmental stage distribution for age and gender is presented in Supplementary Table 1.

**Table 1:** Descriptive statistics of the study population for age, anthropometry, and blood pressure in respect to gender.

**Descriptive variables**	**Gender M/F**	***N***	**Mean**	**SD**	**SE**
Age (years)	M	549	12.04	3.52	0.15
	F	653	12.98	3.83	0.15
SDS height	M	542	0.86	1.03	0.04
	F	647	0.65	1.05	0.04
SDS weight	M	542	0.68	1.09	0.05
	F	647	0.36	1.03	0.04
SDS BMI	M	542	0.31	1.23	0.05
	F	647	0.05	1.08	0.04
SDS waist circumference	M	484	0.67	1.23	0.06
	F	499	0.86	1.27	0.06
Waist to hip circumference ratio	M	544	0.85	0.06	0.00
	F	649	0.82	0.07	0.00
Waist circumference to height ratio	M	538	0.44	0.05	0.00
	F	648	0.44	0.05	0.00
SDS single measurement SBP	M	545	0.93	1.10	0.05
	F	648	0.01	1.27	0.05
SDS single measurement DBP	M	545	0.18	1.49	0.06
	F	648	1.12	1.07	0.04
SDS average SBP	M	401	0.33	1.15	0.06
	F	524	0.10	1.13	0.05
SDS average DBP	M	401	1.23	0.87	0.04
	F	524	1.17	0.90	0.04

*M, male; F, female; BMI, body mass index; SBP, systolic blood pressure; DBP, diastolic blood pressure; SDS, standard deviation score; N, number of subjects*.

Analysis of anthropometric measurements and their indexes (height, weight, BMI, waist circumference, hip circumference, waist to hip circumference ratio, waist circumference to height ratio, and blood pressure) with values above the 95th percentile according to published normative values was done. Obese and hypertensive individuals (*N* = 65) were excluded from the calculation of the normative values. Detailed information on independent variables elevations in the study population is presented in Supplementary Table 2.

### Measurement Variability and Within-Observer Agreement

For intra-observer variability, Pearson correlation coefficients were 0.84 (*p* < 2.2e−16)/0.84(*p* = 1.4e−13)/0.93 (*p* = 5.7e−16) with the mean difference between the measurements (in % of pooled mean cIMT) for individual observer being 0.5–1.5%. Bias and limits of agreement (LOA) are presented in Supplementary Table 3. For the interobserver variability, 75% of compared observer-pairs measurements had correlation coefficients above 0.7 and one third above 0.8, with the mean difference between measurements (in % of pooled mean cIMT) from 1.48 to 6.86%. Bias and limits of agreement for observer-pairs are given in Supplementary Table 4.

### Variables Associated With cIMT

After optimization, the significant multiple linear regression model showed *R*^2^ = 0.12, = *F*_(6;1142df)_ = 26.98, *p* < 0.001. cIMT was lower in girls (β = −7.8, *p* = 0.05), and it increased in both genders with age (β = 18.65, *p* < 0.001), height (β = 5.28, *p* = 0.05), and hip circumference (β = 11.57, *p* < 0.01). The model showed the effect of SDS-BMI on cIMT increase by ~2.6 μm for every unit increase in SDS-hip circumference value. When both BMI and hip circumference were increasing simultaneously, the cIMT increase was even more pronounced.

Linear regression after optimization is summarized in Supplementary Table 5. It was determined by the regression-tree model that age has the largest effect on cIMT deviance. Waist circumference had an important effect on cIMT already at a young age, while BMI became an important factor in adolescence, as presented in Figure 1.

Also, the role of puberty was analyzed. cIMT increased from the pre-pubertal to mid-pubertal and pre-pubertal to late-pubertal stage groups in both genders [ANOVA test result for boys (*F* = 20.503, *p* = 0.000)/for girls (*F* = 9.017, *p* = 0.000), respectively].

### cIMT Reference Values in a Healthy Pediatric Population

**Figure 2:**
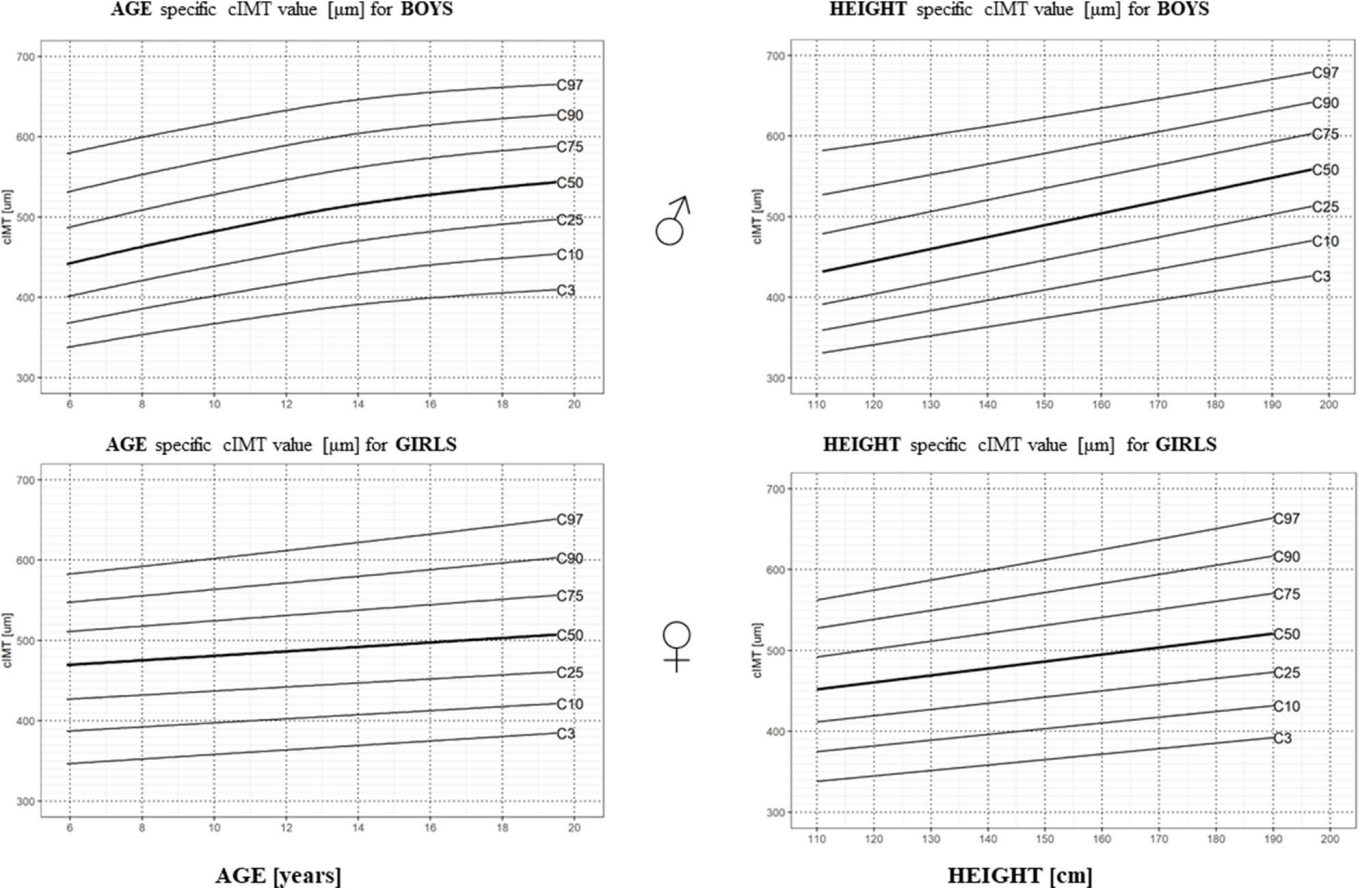
Age- and height-specific centile charts of the right CCA cIMT measured with the RF-QIMT method for boys and girls.

Values of cIMT were normally distributed in the study group after the exclusion of cIMT values > 3SD and <-3SD (The Shapiro–Wilk normality test: *p*-value for male/female study population = 0. 20/0.16). As cIMT differed with gender, age, and height, gender-, age-, and height-specific percentile charts were constructed as presented in Figure 2, in percentile tables in Supplementary Table 6, and gender- and age-specific LMS values in Supplementary Table 7.

### A Systematic Review of Literature on cIMT in a Healthy Pediatric Population

**Figure 3:**
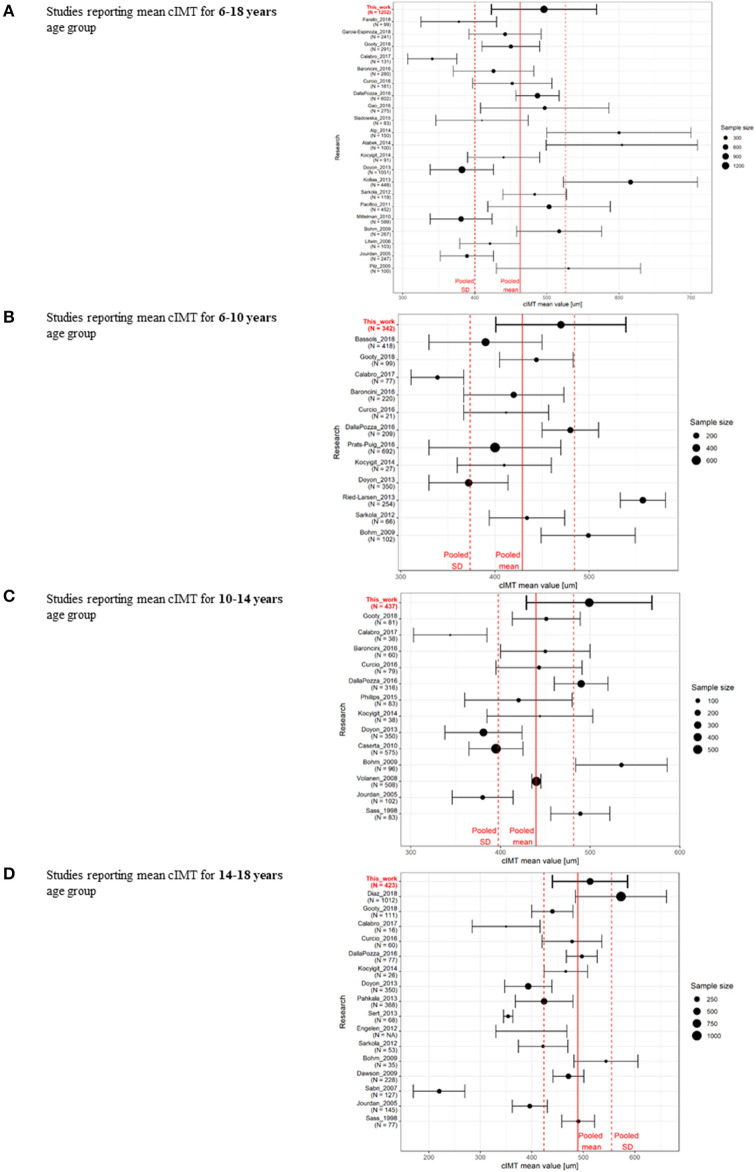
Summary of the review of published data on cIMT in the pediatric population for studies reporting mean cIMT data for **(A)** 6–18-year age range (5, 15–19, 26, 29, 46–58), **(B)** under ten years (5, 16–19, 47, 48, 53, 55, 59–61), **(C)** 10–14 years (16–19, 47, 48, 53, 62–64), **(D)** 14–18-year age range (5, 15, 17–19, 24, 47, 48, 53, 55, 65–70).

Three hundred and twenty-nine articles reporting on cIMT values in the pediatric population were identified; 177 studies that did not separate results for the adult and pediatric population or reported cIMT values for the population with chronic illnesses were excluded. The remaining articles (*n* = 136) were reviewed for reported numerical values of cIMT. After excluding those not reporting age-specific values or had less than ten subjects per year included, 37 studies using manual or semi-automatic measurement techniques were identified for final analysis. The summary of analyzed studies is presented in Figures 3A–D, and in Supplementary Tables 8A–D. cIMT values reported in the present study were within the SD of the pooled data, with a weighted mean value higher than the pooled mean.

## Discussion

The normative cIMT data from the largest single-center cohort of healthy Caucasian children and adolescents aged 6 to 18 years were presented. We used a semiautomatic radio frequency-based, software-guided technique RF-QIMT, which provides real-time image interpretation, suitable for everyday clinical use. This technique was able to detect small differences in cIMT with sufficient sensitivity that allowed for the calculation of gender-, age-, and height-specific normative values for further clinical use in the evaluation of vascular health in high-risk pediatric cohorts of interest.

The absolute cIMT depends on the site of the measurement: it is thicker in the left carotid artery (71) and the carotid bulb (7, 12). The anatomical topography of the carotid bifurcation provides a landmark in imaging but is heterogeneous and adds to the variability of the measurements (36). cIMT varies through the cardiac cycle by as much as 30 μm, being thickest at end diastole and thinnest at peak systole (10). Nonetheless, cIMT values differ due to different ultrasound settings, the intima-media edge detection algorithms, and off-line reading systems (12), with more significant observer-dependent errors in manual measurements (36). Semi-automated edge detection programs have been suggested as a better approach to reduce variability in the measurement of cIMT (31). The reproducibility of measurement in the presented study was demonstrated with acceptable intra-observer variability, and well inside the limits compared to other studies (15, 18, 19, 59) as it reflected the variability of actual separate measurements, contrary to off-line repetitive readings of the same image acquisition. Our study's cIMT measurement technique was fast to perform and well-tolerated in a pediatric population from 6 years of age on, and also in the adolescent age group (19).

The published data are not consistent concerning how cIMT changes during linear growth and pubertal development in children. In the present study, cIMT increased significantly with age, corroborating data from several cross-sectional studies performed with various US methods (5, 7, 15, 19, 72) and contrasting data from studies that failed to demonstrate age-related changes (69). An increase in cIMT is thought to reflect the precedent of vascular aging and the physiological adaptive remodeling of the vessel walls in response to the developmental changes (73). In addition to age, gender does appear to play an essential role in the interpretation of cIMT values in the pediatric population. A steady mean cIMT increase was observed in girls, while in boys, the most significant increase was determined in pre-pubertal years when the annual increase was 10 μm per year, followed by more gradual yearly thickening. A similar pattern of cITM increase was described by Böhm et al. (19), who used semiautomatic US measurement of right CCA on a measurement location 8–18 mm from the bifurcation. cIMT growth chart is gender-specific in pubertal maturing children and adolescents (20); our results contradict some previous studies (10, 15) that failed to detect a significant gender and pubertal maturation influence on cIMT. Pubertal status was in our study obtained by the parent or child report, based on clear instructions, which is shown to be informative (34, 74).

Measures of obesity have a weak correlation with cIMT increase in our study, with SDS-hip circumference and SDS-BMI value having the most significant effect. Of interest, the effect of SDS-hip circumference was determined earlier on SDS-BMI suggesting that determination of hip circumference could be used in risk stratification for AS already in young children. Some previous studies show no correlation between BMI and cIMT under 15 years of age (16), and a positive linear relationship is demonstrated between obesity in childhood and cIMT only in young adults (75, 76). Both absolute and standardized values of systolic blood pressure are related to cIMT, which suggests that blood pressure influences vascular remodeling already in the pediatric population at non-hypertensive levels (77). In the present study, we could not corroborate the results reporting blood pressure influence on cIMT value; this could be due to different cIMT measurement sites.

It is well-known that the ultrasonographic evaluation of cIMT is associated with user-dependent reproducibility errors (8). The main strength of this single medical center study is that it describes a considerably larger cohort compared to previous studies. All the measurements were performed in comparable circumstances by only three examiners trained by the same protocol, which contributed to the lower variability of obtained results. However, it still has to be acknowledged that the US method used has intra-observer and interobserver variability that needs to be considered when using the cIMT marker in clinical practice and decision-making.

Providing normative values for cIMT is challenging, and mean cIMT values may differ considerably between studies (12). A comparison of different cohorts showed high heterogeneity beyond ethnic and geographical factors, limiting the transferability of the results (21, 23), as demonstrated in our comparative analysis of manual and semi-automatic methods alongside the RF-QIMT method of cIMT measurement (Figure 3, Supplementary Tables 8A–D). The cIMT value measured with our technique was within the SD of the pooled data, with a weighted mean value higher from the pooled mean lowered by influential studies using a different technology (5) or site of the measurement (57). Doyon (5) analyzed the most extensive published set of cIMT data in the European pediatric population up to now; however, no racial and ethnic breakdown was provided for this study, and the vast heterogeneity in the methods for cIMT measurement hinders the pooling of their data (78). cIMT was in our study measured only on the right side, and only in CCA; therefore, comparison with some of the studies was limited to a certain degree.

The American Heart Association strongly supports the efforts for AS prevention in youth (4, 30). cIMT is a recognized surrogate biomarker of AS by the American Society of Echocardiography as well as Association for European Pediatric Cardiology (7, 79) that gives noteworthy information on vascular health in the pediatric population, when other markers may not yet mirror vascular alterations. We showed that cIMT was gender-, age-, and height-specific in the pediatric population. Obesity measures influenced its progression.

With providing gender-, age-, and height- specific normative data on mean cIMT in a healthy pediatric population, we propose detecting impaired vascular health from infancy, especially in groups of youth with known risk factors for early AS development, such as diabetes mellitus, obesity, hypercholesterolemia, and low physical fitness, which are gaining in prevalence in the last decades.

cIMT measurement is useful in epidemiological studies (4, 7) including larger study samples; however, in the concept of single-patient follow-up, a degree of variability of measurements should be taken into account when interpreting data, especially in pre-pubertal children. With high-quality gender-, age-, and height-specific normative data on mean cIMT in a healthy pediatric population, we hoped to add to its value and clinical usefulness.

## Data Availability Statement

The raw data supporting the conclusions of this article will be made available by the authors, without undue reservation.

## Ethics Statement

The studies involving human participants were reviewed and approved by The National Medical Ethics Committee approved the study (No. of the approval: 0120-357/2017-/9). Written informed consent to participate in this study was provided by the participants' legal guardian/next of kin.

## Author Contributions

The corresponding author attests that all listed authors meet authorship criteria and that no others meeting the criteria have been omitted. AD and PK participated in the study design and directed the study. AD, PK, and UG conducted the study. PK and UG supervised the project. EP carried out all statistical analyses and provided statistical advice and visual presentations of the results. AD abstracted all the data, wrote the first draft of the paper, and coordinated subsequent revisions. TB and PK will act as guarantors for the paper. All authors contributed to the drafting of the final version of the paper.

## Conflict of Interest

The authors declare that the research was conducted in the absence of any commercial or financial relationships that could be construed as a potential conflict of interest.
