# Supplementary material for: Carotid Intima-Media Thickness in Healthy Children and Adolescents: Normative Data and Systematic Literature Review
Source: Front Cardiovasc Med. 2020 Nov 26;7:597768. doi: 10.3389/fcvm.2020.597768 (PMC7732599; doi:10.3389/fcvm.2020.597768)
Supplement: Supplementary file 1 [file Table_1.DOCX]

**ONLINE SUPPLEMENTAL MATERIAL: Online Figures**

**Online Figure 1:** RF-QIMT based measurement of right common carotid artery far wall imaged in a longitudinal view from a lateral approach. The green box represents the ROI. The green vertical line is placed on bulb origin, the green marker horizontal line is placed in the center of the artery lumen. The green thick horizontal line is superimposed on the B-mode image of the intima-media of the CCA far wall. On the left side of the picture there are the cIMT measurements from the last six cardiac cicles and their averge value. The QIMT value is expresed in micrometers. The numer in the yellow box indicates the standard deviation between measurements.


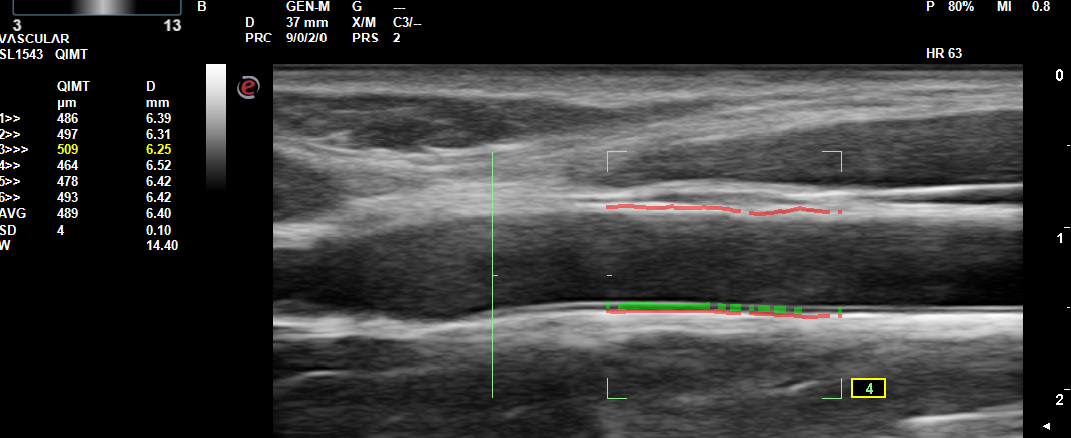


**Online Figure 2:** PRISMA reporting methodology report

**
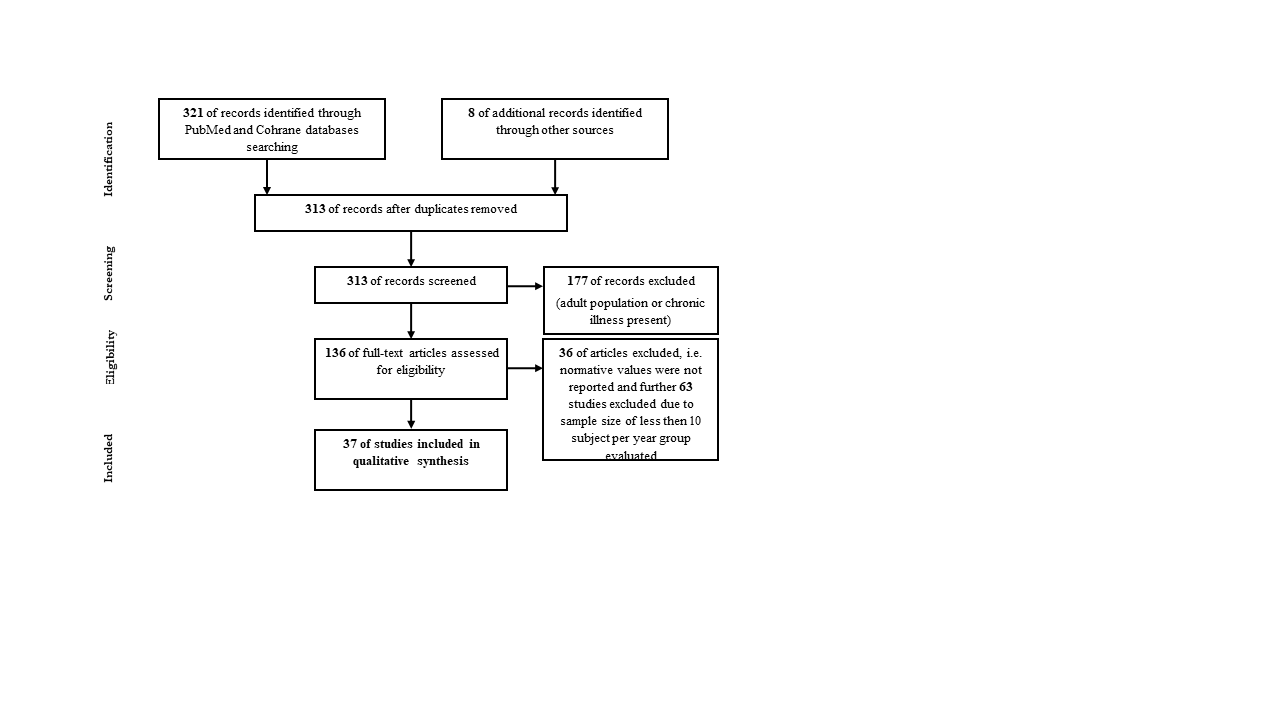
**

**ONLINE SUPPLEMENTAL MATERIAL: Online Tables**

**Online Table 1.** Absolute and relative distributions of age and pubertal developmental stage concerning the gender of the study population.

| Age (years) | Male | | | | Female | | | |
| --- | --- | --- | --- | --- | --- | --- | --- | --- |
|  | Pubertal stage 1  N (%) | Pubertal stage 2  N (%) | Pubertal stage 3  N (%) | Pubertal stage N/A N (%) | Pubertal stage 1  N (%) | Pubertal stage 2  N (%) | Pubertal stage 3  N (%) | Pubertal stage N/A N(%) |
| 6 | 41 (93.2) | 0 (0) | 0 (0) | 3 (6.8) | 37 (100) | 0 (0) | 0 (0) | 0 (0) |
| 7 | 32 (88.9) | 0 (0) | 0 (0) | 4 (11.1) | 25 (78.1) | 0 (0) | 0 (0) | 7 (21.9) |
| 8 | 44 (80) | 2 (3.6) | 0 (0) | 9 (16.4) | 58 (92.1) | 2 (3.2) | 0 (0) | 3 (4.8) |
| 9 | 19 (54.3) | 12 (34.3) | 0 (0) | 4 (11.4) | 30 (75) | 7 (17.5) | 0 (0) | 3 (7.5) |
| 10 | 16 (42.1) | 16 (42.1) | 0 (0) | 6 (15.8) | 15 (45.5) | 10 (30.3) | 4 (12.1) | 4 (12.1) |
| 11 | 32 (33.3) | 47 (49) | 1 (1) | 16 (16.7) | 25 (25.5) | 41 (41.8) | 24 (24.5) | 8 (8.2) |
| 12 | 6 (12) | 27 (54) | 13 (26) | 4 (8) | 2 (4.3) | 17 (36.2) | 24 (51.1) | 4 (8.5) |
| 13 | 8 (20) | 19 (47.5) | 10 (25) | 3 (7.5) | 1 (2.9) | 10 (28.6) | 24 (68.6) | 0 (0) |
| 14 | 1 (5) | 4 (20) | 15 (75) | 0 (0) | 0 (0) | 2 (7.7) | 22 (84.6) | 2 (7.7) |
| 15 | 0 (0) | 3 (9.1) | 27 (81.8) | 3 (9.1) | 0 (0) | 1 (2.3) | 41 (93.2) | 2 (4.5) |
| 16 | 0 (0) | 0 (0) | 41 (97.6) | 1 (2.4) | 0 (0) | 0 (0) | 44 (97.8) | 1 (2.2) |
| 17 | 0 (0) | 0 (0) | 24 (92.3) | 2 (7.7) | 0 (0) | 1 (1.3) | 72 (92.3) | 5 (6.4) |
| 18 | 0 (0) | 1 (3.7) | 25 (92.6) | 1 (3.7) | 0 (0) | 0 (0) | 70 (95.9) | 3 (4.1) |

**Online Table 2:** Absolute and relative prevalence of independent variables elevations between 85^th^ and 95^th^ percentile and above 95^th^ percentile according to different published references.

| Independent variable | Used reference | Gender M/F | Between 85-th and 95-th (%) | Above 95-th (%) |
| --- | --- | --- | --- | --- |
| Percentile height | (Sharma et al., 2015) | M | 123 (22.4) | 114 (20.8) |
|  |  | F | 118 (18.1) | 105 (16.1) |
|  | (KiGGS, 2019) | M | 70 (12.8) | 53 (9.7) |
|  |  | F | 80 (12.3) | 47 (7.2) |
|  | (Jackson et al., 2007) | M | 124 (22.6) | 113 (20.6) |
|  |  | F | 119 (18.2) | 95 (14.5) |
| Percentile weight | (Sharma et al., 2015) | M | 108 (19.7) | 97 (17.7) |
|  |  | F | 90 (13.8) | 75 (11.5) |
|  | (KiGGS, 2019) | M | 57 (10.4) | 28 (5.1) |
|  |  | F | 54 (8.3) | 17 (2.6) |
| Percentile BMI | (Sharma et al., 2015) | M | 74 (13.5) | 72 (13.1) |
|  |  | F | 73 (11.2) | 47 (7.2) |
|  | (KiGGS, 2019) | M | 40 (7.3) | 31 (5.6) |
|  |  | F | 46 (7) | 14 (2.1) |
|  | (Onis et al., 2007) | M | 67 (12.2) | 66 (12) |
|  |  | F | 71 (10.9) | 44 (6.7) |
| Percentile waist circumference | (Sharma et al., 2015) | M | 70 (12.8) | 104 (18.9) |
|  |  | F | 94 (14.4) | 135 (20.7) |
|  | (Rönnecke et al., 2019) | M | 70 (12.8) | 61 (11.1) |
|  |  | F | 95 (14.5) | 74 (11.3) |
|  | (KiGGS, 2019) | M | 34 (6.2) | 25 (4.6) |
|  |  | F | 61 (9.3) | 36 (5.5) |
| Percentile hip circumference | (Rönnecke et al., 2019) | M | 60 (10.9) | 36 (6.6) |
|  |  | F | 53 (8.1) | 25 (3.8) |
|  | (KiGGS, 2019) | M | 31 (5.6) | 12 (2.2) |
|  |  | F | 24 (3.7) | 8 (1.2) |
| Percentile waist to hip circumference ratio | (Rönnecke et al., 2019) | M | 86 (15.7) | 94 (17.1) |
|  |  | F | 108 (16.5) | 164 (25.1) |
|  | (KiGGS, 2019) | M | 49 (8.9) | 40 (7.3) |
|  |  | F | 67 (10.3) | 119 (18.2) |
| Percentile waist circumference to height ratio | (Jackson et al., 2007) | M | 67 (12.2) | 50 (9.1) |
|  |  | F | 93 (14.2) | 72 (11) |
|  | (Sharma et al., 2015) | M | 37 (6.7) | 8 (1.5) |
|  |  | F | 26 (4) | 8 (1.2) |
|  | (KiGGS, 2019) | M | 28 (5.1) | 22 (4) |
|  |  | F | 66 (10.1) | 33 (5.1) |
| Percentile single measurement systolic blood pressure | (Jackson et al., 2007) | M | 122 (22.2) | 114 (20.8) |
|  |  | F | 73 (11.2) | 59 (9) |
|  | (KiGGS, 2019) | M | 88 (16) | 107 (19.5) |
|  |  | F | 104 (15.9) | 140 (21.4) |
| Percentile single measurement dyastolic blood pressure | (Jackson et al., 2007) | M | 69 (12.6) | 98 (17.9) |
|  |  | F | 158 (24.2) | 179 (27.4) |
|  | (KiGGS, 2019) | M | 61 (11.1) | 59 (10.7) |
|  |  | F | 80 (12.3) | 88 (13.5) |
| Percentile average systolic blood pressure | (Jackson et al., 2007) | M | 58 (10.6) | 54 (9.8) |
|  |  | F | 77 (11.8) | 36 (5.5) |
|  | (KiGGS, 2019) | M | 84 (15.3) | 88 (16) |
|  |  | F | 86 (13.2) | 121 (18.5) |
| Percentile average dyastolic blood pressure | (Jackson et al., 2007) | M | 94 (17.1) | 130 (23.7) |
|  |  | F | 150 (23) | 149 (22.8) |
|  | (KiGGS, 2019) | M | 0 (0) | 400 (72.9) |
|  |  | F | 0 (0) | 528 (80.9) |

**References:**

- FOR EP, CHILDREN RR. Expert panel on integrated guidelines for cardiovascular health and risk reduction in children and adolescents: summary report. Pediatrics 2011;128(5):S213.
- Jackson LV, Thalange NK, Cole TJ. Blood pressure centiles for Great Britain. Arch Dis Child 2007;92(4):298–303.
- Neuhauser HK, Thamm M, Ellert U, et al. Blood Pressure Percentiles by Age and Height From Nonoverweight Children and Adolescents in Germany. Pediatrics 2011;127(4):e978–88.
- Onis MD, Onyango AW, Borghi E, et al. Development of a WHO growth reference for school-aged children and adolescents. Bulletin of the World health Organization. 2007;85:660-7.
- Rönnecke E, Vogel M, Bussler S, et al. Age- and Sex-Related Percentiles of Skinfold Thickness, Waist and Hip Circumference, Waist-to-Hip Ratio and Waist-to-Height Ratio: Results from a Population-Based Pediatric Cohort in Germany (LIFE Child). Obes Facts 2019;12(1):25–39.
- Sharma AK, Metzger DL, Daymont C, et al. LMS tables for waist-circumference and waist-height ratio Z-scores in children aged 5–19 y in NHANES III: Association with cardio-metabolic risks. Pediatr Res 2015;78(6):723–9.

**Online Table 3:** Bias and limit of agreement for intra-observer analysis

| Comparison | Bias | Lower LOA | Upper LOA |
| --- | --- | --- | --- |
| A1 vs A2 | 5.173333 | -65.80580 | 76.15247 |
| P1 vs P2 | 7.446809 | -81.58798 | 96.48159 |
| U1 vs U2 | 2.342857 | -47.22557 | 51.91129 |

**Legend:** LOA- limit of agreement; A1-observer 1, measurement 1; A2-observer 1, measurement 2; P1-observer 2, measurement 1; P2-observer 2, measurement 2; U1-observer 3, measurement 1; U2-observer 3, measurement 2;

**Online Table 4:** Bias and limit of agreement LOA for inter-observer analysis

| Comparison | Bias | LowerLOA | UpperLOA |
| --- | --- | --- | --- |
| A1 vs P1 | -33.697674 | -168.22670 | 100.83135 |
| A1 vs P2 | -32.813953 | -143.38037 | 77.75246 |
| A1 vs U1 | -11.675676 | -105.83057 | 82.47922 |
| A1 vs U2 | -7.371429 | -106.68287 | 91.94001 |
| A2 vs P1 | -35.682927 | -134.55171 | 63.18585 |
| A2 vs P2 | -33.926829 | -115.66422 | 47.81056 |
| A2 vs U1 | -26.571429 | -118.84007 | 65.69721 |
| A2 vs U2 | -15.090909 | -100.54129 | 70.35948 |
| P1 vs U1 | 9.000000 | -80.70436 | 98.70436 |
| P1 vs U2 | 13.857143 | -56.13278 | 83.84706 |
| P2 vs U1 | 10.714286 | -58.61649 | 80.04506 |
| P2 vs U2 | 15.571429 | -43.94136 | 75.08422 |

**Legend:** LOA- limit of agreement; A1-observer 1, measurement 1; A2-observer 1, measurement 2; P1-observer 2, measurement 1; P2-observer 2, measurement 2; U1-observer 3, measurement 1; U2-observer 3, measurement 2;

| Formula | cIMT ~ gender + SDS_age + SDS_height + SDS_hip circumference + SDS-BMI:SDS-hip circumferecnce |
| --- | --- |
| Residuals | Min 1Q Median 3Q Max  -192,9 -44.03 -2.69 43.89 191.263 |
| Coefficients | \|  \| Estimate \| SE \| T value \| Pr(>\|t\|) \| Significance codes: \| \| --- \| --- \| --- \| --- \| --- \| --- \| \| Intercept \| 492.45 \| 3.26 \| 150.872 \| < 2e-16 \| *** \| \| Female gender \| -7.77 \| 3.99 \| -1.948 \| 0.05 \|  \| \| Age \| 18.65 \| 1.98 \| 9.407 \| < 2e-16 \| *** \| \| SDS-height \| 5.28 \| 2.38 \| 2.22 \| 0.027 \| * \| \| SDS_hip circumference \| 11.57 \| 4.37 \| 2.65 \| 0.008 \| ** \| \| SDS-BMI: SDS-hip circumference \| 2.6 \| 1.62 \| 1.6 \| 0.1 \|  \| |
| Residual SE | 65.32 on 1142 df |
| Multiple R ^ 2 | 0.1242 |
| Adjusted R^ 2 | 0.1196 |
| F-statistic | 26.98 on 6 and 1142 df, p-value: < 2.2e-16 |

**Online Table 5.** Linear regression model characteristics.

Significance codes: 0 '***' 0.001 '**' 0.01 '*' 0.05 '.' 0.1 ' ' 1; SE standard error, R ^ 2 R squared, df degrees of freedom

**Online Table 6: Age (A, B) and height (C, D) specific percentile values of cIMT (µm) for boys (A, C) and for girls (B, D).**

**A Age specific percentile values of cIMT (µm) for boys**

| Age (years) | C5 | C10 | C25 | **C50** | C75 | C90 | **C95** |
| --- | --- | --- | --- | --- | --- | --- | --- |
| 6 | 349 | 368 | 401 | **442** | 487 | 532 | **560** |
| 7 | 357 | 377 | 411 | **452** | 498 | 542 | **570** |
| 8 | 365 | 385 | 421 | **463** | 508 | 552 | **580** |
| 9 | 373 | 393 | 430 | **472** | 518 | 562 | **590** |
| 10 | 380 | 401 | 438 | **482** | 528 | 571 | **598** |
| 11 | 387 | 409 | 447 | **491** | 537 | 580 | **607** |
| 12 | 394 | 416 | 455 | **500** | 546 | 589 | **615** |
| 13 | 400 | 423 | 463 | **508** | 554 | 597 | **623** |
| 14 | 406 | 430 | 470 | **515** | 562 | 604 | **629** |
| 15 | 411 | 435 | 476 | **522** | 568 | 610 | **635** |
| 16 | 415 | 440 | 481 | **527** | 573 | 614 | **639** |
| 17 | 419 | 444 | 486 | **532** | 578 | 619 | **643** |
| 18 | 422 | 448 | 491 | **537** | 582 | 622 | **646** |

**B Age specific percentile values of cIMT (µm) for girls**

| Age (years) | C5 | C10 | C25 | **C50** | C75 | C90 | **C95** |
| --- | --- | --- | --- | --- | --- | --- | --- |
| 6 | 362 | 387 | 427 | **469** | 511 | 547 | **569** |
| 7 | 365 | 389 | 429 | **472** | 514 | 551 | **573** |
| 8 | 368 | 392 | 432 | **475** | 517 | 555 | **578** |
| 9 | 370 | 394 | 434 | **478** | 521 | 559 | **582** |
| 10 | 373 | 397 | 437 | **480** | 524 | 563 | **586** |
| 11 | 376 | 399 | 439 | **483** | 527 | 567 | **591** |
| 12 | 378 | 402 | 442 | **486** | 531 | 571 | **596** |
| 13 | 381 | 405 | 444 | **489** | 534 | 575 | **600** |
| 14 | 384 | 407 | 447 | **492** | 537 | 579 | **605** |
| 15 | 386 | 410 | 449 | **494** | 541 | 584 | **610** |
| 16 | 389 | 412 | 452 | **497** | 544 | 588 | **614** |
| 17 | 392 | 415 | 454 | **500** | 547 | 592 | **619** |
| 18 | 394 | 417 | 457 | **503** | 551 | 596 | **624** |

**C Height specific percentile values of cIMT (µm) for boys**

| Height (cm) | C5 | C10 | C25 | **C50** | C75 | C90 | **C95** |
| --- | --- | --- | --- | --- | --- | --- | --- |
| 111 | 341 | 359 | 391 | **431** | 478 | 527 | **559** |
| 112 | 342 | 360 | 392 | **433** | 480 | 528 | **560** |
| 113 | 344 | 361 | 394 | **434** | 481 | 529 | **561** |
| 114 | 345 | 363 | 395 | **436** | 483 | 531 | **562** |
| 115 | 346 | 364 | 396 | **437** | 484 | 532 | **564** |
| 116 | 347 | 365 | 398 | **439** | 486 | 533 | **565** |
| 117 | 348 | 366 | 399 | **440** | 487 | 535 | **566** |
| 118 | 349 | 368 | 401 | **442** | 488 | 536 | **567** |
| 119 | 351 | 369 | 402 | **443** | 490 | 537 | **568** |
| 120 | 352 | 370 | 403 | **445** | 491 | 538 | **569** |
| 121 | 353 | 371 | 405 | **446** | 493 | 540 | **570** |
| 122 | 354 | 373 | 406 | **448** | 494 | 541 | **572** |
| 123 | 355 | 374 | 408 | **449** | 496 | 542 | **573** |
| 124 | 357 | 375 | 409 | **451** | 497 | 544 | **574** |
| 125 | 358 | 376 | 410 | **452** | 499 | 545 | **575** |
| 126 | 359 | 378 | 412 | **454** | 500 | 546 | **576** |
| 127 | 360 | 379 | 413 | **455** | 501 | 548 | **577** |
| 128 | 361 | 380 | 415 | **457** | 503 | 549 | **578** |
| 129 | 362 | 382 | 416 | **458** | 504 | 550 | **580** |
| 130 | 364 | 383 | 417 | **459** | 506 | 552 | **581** |
| 131 | 365 | 384 | 419 | **461** | 507 | 553 | **582** |
| 132 | 366 | 385 | 420 | **462** | 509 | 554 | **583** |
| 133 | 367 | 387 | 422 | **464** | 510 | 555 | **584** |
| 134 | 368 | 388 | 423 | **465** | 512 | 557 | **586** |
| 135 | 369 | 389 | 424 | **467** | 513 | 558 | **587** |
| 136 | 371 | 391 | 426 | **468** | 514 | 559 | **588** |
| 137 | 372 | 392 | 427 | **470** | 516 | 561 | **589** |
| 138 | 373 | 393 | 429 | **471** | 517 | 562 | **590** |
| 139 | 374 | 394 | 430 | **473** | 519 | 563 | **592** |
| 140 | 375 | 396 | 431 | **474** | 520 | 565 | **593** |
| 141 | 377 | 397 | 433 | **476** | 522 | 566 | **594** |
| 142 | 378 | 398 | 434 | **477** | 523 | 567 | **595** |
| 143 | 379 | 399 | 436 | **479** | 525 | 569 | **596** |
| 144 | 380 | 401 | 437 | **480** | 526 | 570 | **598** |
| 145 | 381 | 402 | 439 | **482** | 528 | 571 | **599** |
| 146 | 382 | 403 | 440 | **483** | 529 | 573 | **600** |
| 147 | 384 | 405 | 441 | **485** | 530 | 574 | **601** |
| 148 | 385 | 406 | 443 | **486** | 532 | 575 | **602** |
| 149 | 386 | 407 | 444 | **487** | 533 | 577 | **604** |
| 150 | 387 | 408 | 446 | **489** | 535 | 578 | **605** |
| 151 | 388 | 410 | 447 | **490** | 536 | 579 | **606** |
| 152 | 390 | 411 | 448 | **492** | 538 | 581 | **607** |
| 153 | 391 | 412 | 450 | **493** | 539 | 582 | **609** |
| 154 | 392 | 414 | 451 | **495** | 541 | 583 | **610** |
| 155 | 393 | 415 | 453 | **496** | 542 | 585 | **611** |
| 156 | 394 | 416 | 454 | **498** | 543 | 586 | **612** |
| 157 | 396 | 418 | 455 | **499** | 545 | 587 | **613** |
| 158 | 397 | 419 | 457 | **501** | 546 | 589 | **615** |
| 159 | 398 | 420 | 458 | **502** | 548 | 590 | **616** |
| 160 | 399 | 421 | 460 | **504** | 549 | 591 | **617** |
| 161 | 400 | 423 | 461 | **505** | 551 | 593 | **618** |
| 162 | 401 | 424 | 463 | **507** | 552 | 594 | **620** |
| 163 | 403 | 425 | 464 | **508** | 554 | 595 | **621** |
| 164 | 404 | 427 | 465 | **510** | 555 | 597 | **622** |
| 165 | 405 | 428 | 467 | **511** | 556 | 598 | **623** |
| 166 | 406 | 429 | 468 | **513** | 558 | 599 | **625** |
| 167 | 407 | 430 | 470 | **514** | 559 | 601 | **626** |
| 168 | 409 | 432 | 471 | **516** | 561 | 602 | **627** |
| 169 | 410 | 433 | 472 | **517** | 562 | 603 | **628** |
| 170 | 411 | 434 | 474 | **518** | 564 | 605 | **630** |
| 171 | 412 | 436 | 475 | **520** | 565 | 606 | **631** |
| 172 | 413 | 437 | 477 | **521** | 567 | 608 | **632** |
| 173 | 415 | 438 | 478 | **523** | 568 | 609 | **634** |
| 174 | 416 | 440 | 480 | **524** | 569 | 610 | **635** |
| 175 | 417 | 441 | 481 | **526** | 571 | 612 | **636** |
| 176 | 418 | 442 | 482 | **527** | 572 | 613 | **637** |
| 177 | 419 | 444 | 484 | **529** | 574 | 614 | **639** |
| 178 | 421 | 445 | 485 | **530** | 575 | 616 | **640** |
| 179 | 422 | 446 | 487 | **532** | 577 | 617 | **641** |
| 180 | 423 | 447 | 488 | **533** | 578 | 618 | **642** |
| 181 | 424 | 449 | 490 | **535** | 580 | 620 | **644** |
| 182 | 425 | 450 | 491 | **536** | 581 | 621 | **645** |
| 183 | 427 | 451 | 492 | **538** | 582 | 622 | **646** |
| 184 | 428 | 453 | 494 | **539** | 584 | 624 | **647** |
| 185 | 429 | 454 | 495 | **541** | 585 | 625 | **649** |
| 186 | 430 | 455 | 497 | **542** | 587 | 626 | **650** |
| 187 | 431 | 457 | 498 | **544** | 588 | 628 | **651** |
| 188 | 433 | 458 | 500 | **545** | 590 | 629 | **653** |
| 189 | 434 | 459 | 501 | **546** | 591 | 631 | **654** |
| 190 | 435 | 461 | 502 | **548** | 593 | 632 | **655** |

**D Height specific percentile values of cIMT (µm) for girls**

| Height (cm) | C5 | C10 | C25 | **C50** | C75 | C90 | **C95** |
| --- | --- | --- | --- | --- | --- | --- | --- |
| 111 | 353 | 375 | 412 | **452** | 492 | 528 | **549** |
| 112 | 353 | 376 | 413 | **453** | 493 | 529 | **551** |
| 113 | 354 | 376 | 413 | **454** | 494 | 530 | **552** |
| 114 | 355 | 377 | 414 | **455** | 495 | 531 | **553** |
| 115 | 355 | 378 | 415 | **456** | 496 | 532 | **554** |
| 116 | 356 | 379 | 416 | **457** | 497 | 534 | **555** |
| 117 | 357 | 379 | 416 | **458** | 498 | 535 | **556** |
| 118 | 358 | 380 | 417 | **458** | 499 | 536 | **558** |
| 119 | 358 | 381 | 418 | **459** | 500 | 537 | **559** |
| 120 | 359 | 381 | 419 | **460** | 501 | 538 | **560** |
| 121 | 360 | 382 | 420 | **461** | 502 | 539 | **561** |
| 122 | 360 | 383 | 420 | **462** | 503 | 540 | **562** |
| 123 | 361 | 384 | 421 | **463** | 504 | 541 | **563** |
| 124 | 362 | 384 | 422 | **464** | 505 | 542 | **565** |
| 125 | 362 | 385 | 423 | **464** | 506 | 543 | **566** |
| 126 | 363 | 386 | 423 | **465** | 507 | 545 | **567** |
| 127 | 364 | 386 | 424 | **466** | 508 | 546 | **568** |
| 128 | 364 | 387 | 425 | **467** | 509 | 547 | **569** |
| 129 | 365 | 388 | 426 | **468** | 510 | 548 | **571** |
| 130 | 366 | 388 | 427 | **469** | 511 | 549 | **572** |
| 131 | 366 | 389 | 427 | **470** | 512 | 550 | **573** |
| 132 | 367 | 390 | 428 | **470** | 513 | 551 | **574** |
| 133 | 368 | 391 | 429 | **471** | 514 | 552 | **575** |
| 134 | 368 | 391 | 430 | **472** | 515 | 553 | **576** |
| 135 | 369 | 392 | 430 | **473** | 516 | 555 | **578** |
| 136 | 370 | 393 | 431 | **474** | 517 | 556 | **579** |
| 137 | 371 | 393 | 432 | **475** | 518 | 557 | **580** |
| 138 | 371 | 394 | 433 | **476** | 519 | 558 | **581** |
| 139 | 372 | 395 | 433 | **477** | 520 | 559 | **582** |
| 140 | 373 | 396 | 434 | **477** | 521 | 560 | **584** |
| 141 | 373 | 396 | 435 | **478** | 522 | 561 | **585** |
| 142 | 374 | 397 | 436 | **479** | 523 | 562 | **586** |
| 143 | 375 | 398 | 437 | **480** | 524 | 563 | **587** |
| 144 | 375 | 398 | 437 | **481** | 525 | 565 | **588** |
| 145 | 376 | 399 | 438 | **482** | 526 | 566 | **590** |
| 146 | 377 | 400 | 439 | **483** | 527 | 567 | **591** |
| 147 | 377 | 401 | 440 | **483** | 528 | 568 | **592** |
| 148 | 378 | 401 | 440 | **484** | 529 | 569 | **593** |
| 149 | 379 | 402 | 441 | **485** | 530 | 570 | **594** |
| 150 | 379 | 403 | 442 | **486** | 531 | 571 | **596** |
| 151 | 380 | 403 | 443 | **487** | 532 | 572 | **597** |
| 152 | 381 | 404 | 443 | **488** | 533 | 573 | **598** |
| 153 | 382 | 405 | 444 | **489** | 534 | 575 | **599** |
| 154 | 382 | 406 | 445 | **489** | 535 | 576 | **600** |
| 155 | 383 | 406 | 446 | **490** | 536 | 577 | **602** |
| 156 | 384 | 407 | 447 | **491** | 537 | 578 | **603** |
| 157 | 384 | 408 | 447 | **492** | 537 | 579 | **604** |
| 158 | 385 | 408 | 448 | **493** | 538 | 580 | **605** |
| 159 | 386 | 409 | 449 | **494** | 539 | 581 | **607** |
| 160 | 386 | 410 | 450 | **495** | 540 | 582 | **608** |
| 161 | 387 | 411 | 450 | **495** | 541 | 584 | **609** |
| 162 | 388 | 411 | 451 | **496** | 542 | 585 | **610** |
| 163 | 388 | 412 | 452 | **497** | 543 | 586 | **611** |
| 164 | 389 | 413 | 453 | **498** | 544 | 587 | **613** |
| 165 | 390 | 413 | 453 | **499** | 545 | 588 | **614** |
| 166 | 391 | 414 | 454 | **500** | 546 | 589 | **615** |
| 167 | 391 | 415 | 455 | **501** | 547 | 590 | **616** |
| 168 | 392 | 416 | 456 | **502** | 548 | 591 | **618** |
| 169 | 393 | 416 | 457 | **502** | 549 | 593 | **619** |
| 170 | 393 | 417 | 457 | **503** | 550 | 594 | **620** |
| 171 | 394 | 418 | 458 | **504** | 551 | 595 | **621** |
| 172 | 395 | 418 | 459 | **505** | 552 | 596 | **622** |
| 173 | 395 | 419 | 460 | **506** | 553 | 597 | **624** |
| 174 | 396 | 420 | 460 | **507** | 554 | 598 | **625** |
| 175 | 397 | 420 | 461 | **508** | 555 | 599 | **626** |
| 176 | 397 | 421 | 462 | **508** | 556 | 600 | **627** |
| 177 | 398 | 422 | 463 | **509** | 557 | 602 | **629** |
| 178 | 399 | 423 | 463 | **510** | 558 | 603 | **630** |
| 179 | 399 | 423 | 464 | **511** | 559 | 604 | **631** |
| 180 | 400 | 424 | 465 | **512** | 560 | 605 | **632** |
| 181 | 401 | 425 | 466 | **513** | 561 | 606 | **634** |
| 182 | 402 | 425 | 466 | **514** | 562 | 607 | **635** |
| 183 | 402 | 426 | 467 | **514** | 563 | 608 | **636** |
| 184 | 403 | 427 | 468 | **515** | 564 | 610 | **637** |
| 185 | 404 | 428 | 469 | **516** | 565 | 611 | **639** |

**Online Table 7:** **Gender, age (A) and height (B) specific LMS (the mean (M), the coefficient of variation (S), and the measure of skewness (L)) values of cIMT measured with RF- QIMT method.**

**A Age-specific LMS table of cIMT (µm) for boys and girls**

|  | **Boys** | | | **Girls** | | |
| --- | --- | --- | --- | --- | --- | --- |
| **Age (years)** | **L** | **M** | **S** | **L** | **M** | **S** |
| 6 | -0.034 | 442 | 0.143 | 1.331 | 469 | 0.133 |
| 6.5 | 0.020 | 447 | 0.143 | 1.296 | 471 | 0.133 |
| 7 | 0.073 | 452 | 0.142 | 1.261 | 472 | 0.133 |
| 7.5 | 0.126 | 458 | 0.141 | 1.227 | 473 | 0.134 |
| 8 | 0.180 | 463 | 0.140 | 1.192 | 475 | 0.134 |
| 8.5 | 0.233 | 468 | 0.140 | 1.157 | 476 | 0.134 |
| 9 | 0.286 | 472 | 0.139 | 1.122 | 478 | 0.134 |
| 9.5 | 0.340 | 477 | 0.138 | 1.087 | 479 | 0.135 |
| 10 | 0.393 | 482 | 0.137 | 1.053 | 480 | 0.135 |
| 10.5 | 0.446 | 486 | 0.137 | 1.018 | 482 | 0.135 |
| 11 | 0.500 | 491 | 0.136 | 0.983 | 483 | 0.135 |
| 11.5 | 0.553 | 495 | 0.135 | 0.948 | 485 | 0.136 |
| 12 | 0.606 | 500 | 0.135 | 0.913 | 486 | 0.136 |
| 12.5 | 0.660 | 504 | 0.134 | 0.878 | 487 | 0.136 |
| 13 | 0.713 | 508 | 0.133 | 0.844 | 489 | 0.136 |
| 13.5 | 0.766 | 512 | 0.132 | 0.809 | 490 | 0.137 |
| 14 | 0.820 | 515 | 0.132 | 0.774 | 492 | 0.137 |
| 14.5 | 0.873 | 519 | 0.131 | 0.739 | 493 | 0.137 |
| 15 | 0.926 | 522 | 0.130 | 0.704 | 494 | 0.137 |
| 15.5 | 0.980 | 525 | 0.130 | 0.670 | 496 | 0.138 |
| 16 | 1.033 | 527 | 0.129 | 0.635 | 497 | 0.138 |
| 16.5 | 1.086 | 530 | 0.128 | 0.600 | 498 | 0.138 |
| 17 | 1.140 | 532 | 0.128 | 0.565 | 500 | 0.138 |
| 17.5 | 1.193 | 535 | 0.127 | 0.530 | 501 | 0.139 |
| 18 | 1.246 | 537 | 0.126 | 0.495 | 503 | 0.139 |

**B Height-specific LMS table of cIMT (µm) for boys and girls**

|  | **Boys** | | | **Girls** | | |
| --- | --- | --- | --- | --- | --- | --- |
| **Height (cm)** | **L** | **M** | **S** | **L** | **M** | **S** |
| 111 | -0.405 | 431 | 0.149 | 1.122 | 452 | 0.132 |
| 112 | -0.384 | 433 | 0.149 | 1.115 | 453 | 0.132 |
| 113 | -0.363 | 434 | 0.149 | 1.109 | 454 | 0.132 |
| 114 | -0.342 | 436 | 0.148 | 1.103 | 455 | 0.132 |
| 115 | -0.321 | 437 | 0.148 | 1.096 | 456 | 0.132 |
| 116 | -0.299 | 439 | 0.148 | 1.090 | 457 | 0.132 |
| 117 | -0.278 | 440 | 0.147 | 1.083 | 458 | 0.132 |
| 118 | -0.257 | 442 | 0.147 | 1.077 | 458 | 0.132 |
| 119 | -0.236 | 443 | 0.146 | 1.070 | 459 | 0.132 |
| 120 | -0.215 | 445 | 0.146 | 1.064 | 460 | 0.133 |
| 121 | -0.194 | 446 | 0.146 | 1.057 | 461 | 0.133 |
| 122 | -0.173 | 448 | 0.145 | 1.051 | 462 | 0.133 |
| 123 | -0.152 | 449 | 0.145 | 1.044 | 463 | 0.133 |
| 124 | -0.131 | 451 | 0.144 | 1.038 | 464 | 0.133 |
| 125 | -0.110 | 452 | 0.144 | 1.032 | 464 | 0.133 |
| 126 | -0.089 | 454 | 0.144 | 1.025 | 465 | 0.133 |
| 127 | -0.068 | 455 | 0.143 | 1.019 | 466 | 0.133 |
| 128 | -0.047 | 457 | 0.143 | 1.012 | 467 | 0.133 |
| 129 | -0.026 | 458 | 0.143 | 1.006 | 468 | 0.133 |
| 130 | -0.005 | 459 | 0.142 | 0.999 | 469 | 0.133 |
| 131 | 0.016 | 461 | 0.142 | 0.993 | 470 | 0.134 |
| 132 | 0.037 | 462 | 0.142 | 0.986 | 470 | 0.134 |
| 133 | 0.058 | 464 | 0.141 | 0.980 | 471 | 0.134 |
| 134 | 0.079 | 465 | 0.141 | 0.973 | 472 | 0.134 |
| 135 | 0.100 | 467 | 0.140 | 0.967 | 473 | 0.134 |
| 136 | 0.121 | 468 | 0.140 | 0.961 | 474 | 0.134 |
| 137 | 0.142 | 470 | 0.140 | 0.954 | 475 | 0.134 |
| 138 | 0.163 | 471 | 0.139 | 0.948 | 476 | 0.134 |
| 139 | 0.184 | 473 | 0.139 | 0.941 | 477 | 0.134 |
| 140 | 0.205 | 474 | 0.139 | 0.935 | 477 | 0.134 |
| 141 | 0.226 | 476 | 0.138 | 0.928 | 478 | 0.134 |
| 142 | 0.247 | 477 | 0.138 | 0.922 | 479 | 0.134 |
| 143 | 0.268 | 479 | 0.138 | 0.915 | 480 | 0.135 |
| 144 | 0.289 | 480 | 0.137 | 0.908 | 481 | 0.135 |
| 145 | 0.310 | 482 | 0.137 | 0.902 | 482 | 0.135 |
| 146 | 0.331 | 483 | 0.136 | 0.895 | 483 | 0.135 |
| 147 | 0.352 | 485 | 0.136 | 0.889 | 483 | 0.135 |
| 148 | 0.374 | 486 | 0.136 | 0.882 | 484 | 0.135 |
| 149 | 0.395 | 487 | 0.135 | 0.876 | 485 | 0.135 |
| 150 | 0.416 | 489 | 0.135 | 0.869 | 486 | 0.135 |
| 151 | 0.437 | 490 | 0.135 | 0.862 | 487 | 0.135 |
| 152 | 0.458 | 492 | 0.134 | 0.856 | 488 | 0.135 |
| 153 | 0.479 | 493 | 0.134 | 0.849 | 489 | 0.135 |
| 154 | 0.500 | 495 | 0.134 | 0.842 | 489 | 0.136 |
| 155 | 0.521 | 496 | 0.133 | 0.836 | 490 | 0.136 |
| 156 | 0.542 | 498 | 0.133 | 0.829 | 491 | 0.136 |
| 157 | 0.563 | 499 | 0.133 | 0.822 | 492 | 0.136 |
| 158 | 0.584 | 501 | 0.132 | 0.816 | 493 | 0.136 |
| 159 | 0.605 | 502 | 0.132 | 0.809 | 494 | 0.136 |
| 160 | 0.626 | 504 | 0.132 | 0.802 | 495 | 0.136 |
| 161 | 0.647 | 505 | 0.131 | 0.796 | 495 | 0.136 |
| 162 | 0.668 | 507 | 0.131 | 0.789 | 496 | 0.136 |
| 163 | 0.689 | 508 | 0.131 | 0.782 | 497 | 0.136 |
| 164 | 0.710 | 510 | 0.130 | 0.775 | 498 | 0.136 |
| 165 | 0.731 | 511 | 0.130 | 0.769 | 499 | 0.137 |
| 166 | 0.752 | 513 | 0.130 | 0.762 | 500 | 0.137 |
| 167 | 0.773 | 514 | 0.129 | 0.755 | 501 | 0.137 |
| 168 | 0.794 | 516 | 0.129 | 0.749 | 502 | 0.137 |
| 169 | 0.815 | 517 | 0.129 | 0.742 | 502 | 0.137 |
| 170 | 0.836 | 518 | 0.128 | 0.735 | 503 | 0.137 |
| 171 | 0.857 | 520 | 0.128 | 0.728 | 504 | 0.137 |
| 172 | 0.878 | 521 | 0.128 | 0.722 | 505 | 0.137 |
| 173 | 0.899 | 523 | 0.127 | 0.715 | 506 | 0.137 |
| 174 | 0.920 | 524 | 0.127 | 0.708 | 507 | 0.137 |
| 175 | 0.941 | 526 | 0.127 | 0.701 | 508 | 0.137 |
| 176 | 0.962 | 527 | 0.126 | 0.695 | 508 | 0.138 |
| 177 | 0.983 | 529 | 0.126 | 0.688 | 509 | 0.138 |
| 178 | 1.004 | 530 | 0.126 | 0.681 | 510 | 0.138 |
| 179 | 1.025 | 532 | 0.125 | 0.674 | 511 | 0.138 |
| 180 | 1.046 | 533 | 0.125 | 0.668 | 512 | 0.138 |
| 181 | 1.068 | 535 | 0.125 | 0.661 | 513 | 0.138 |
| 182 | 1.089 | 536 | 0.124 | 0.654 | 514 | 0.138 |
| 183 | 1.110 | 538 | 0.124 | 0.647 | 514 | 0.138 |
| 184 | 1.131 | 539 | 0.124 | 0.641 | 515 | 0.138 |
| 185 | 1.152 | 541 | 0.123 | 0.634 | 516 | 0.138 |
| 186 | 1.173 | 542 | 0.123 | 0.627 | 517 | 0.138 |
| 187 | 1.194 | 544 | 0.123 | 0.620 | 518 | 0.139 |
| 188 | 1.215 | 545 | 0.122 | 0.614 | 519 | 0.139 |
| 189 | 1.236 | 546 | 0.122 | 0.607 | 520 | 0.139 |
| 190 | 1.257 | 548 | 0.122 | 0.600 | 520 | 0.139 |

**Online Table 8:** Summary of the Review of published data on manual, semi-automatic and RF-QIMT methods of cIMT measurement in the pediatric population. Mean cIMT obtained in our and other published studies divided into age groups for comparison: **A** 6-20 years, **B** under ten years, **C** 10- 14 years, and **D** 14-18 years of age.

**Legend: Site of the measurement on common carotid arthery(CCA):**

A= 1 cm from the bulb

B= 1 cm from the bifurcation

C= 1-2 cm from the bifurcation

D= near the bifurcation

E= 8-18 mm from the bifurcation

F= at the bulb

G= 1-2 cm from the bulb

H= 0,5-3 cm from the bifurcation

R= right CCA

L=left CCA

| **A: Age group 6-18 years** | | | |
| --- | --- | --- | --- |
| **Research** | **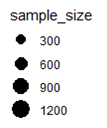Pooled mean cIMT (µm) and SD** | **Reported device and method of cIMT measurement** | **Site of measurement on the CCA** |
| 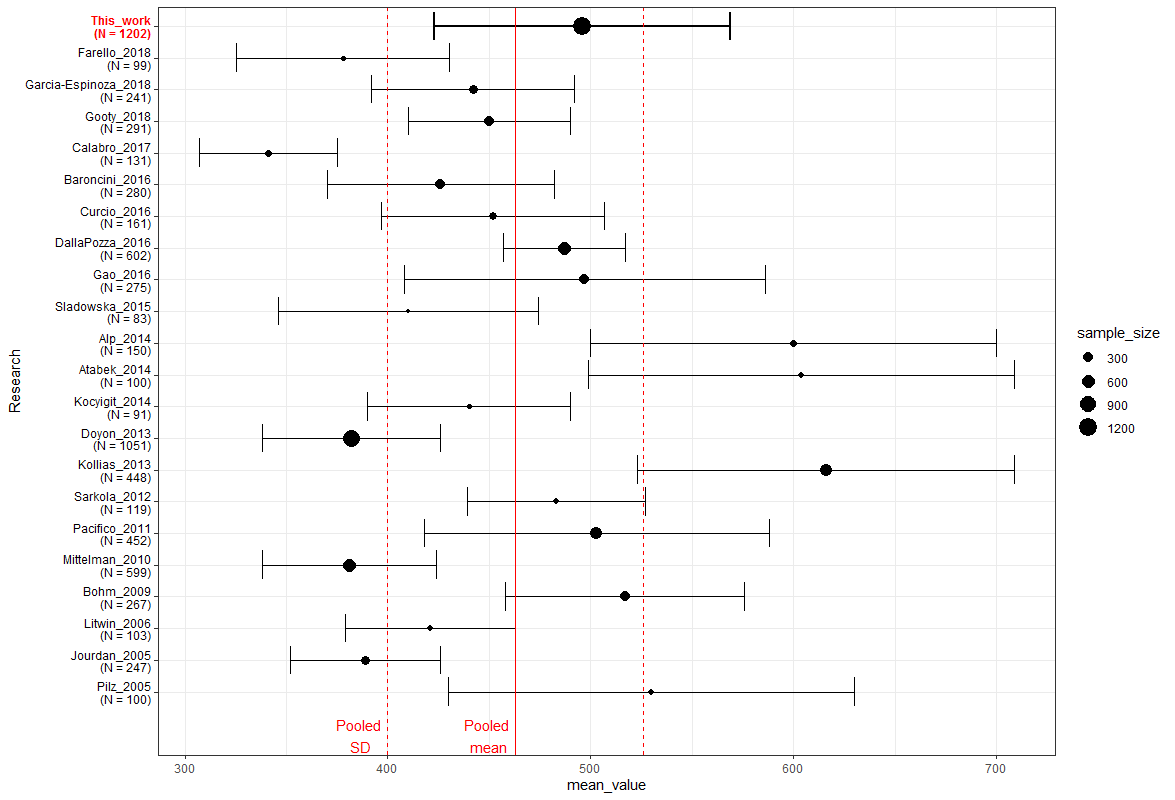 | | RF-QIMT, Esaote, Italy; S.A.  Color Doppler, Technos MPX Esaote, Italy; S.A.  Hemodyn-4 M, Dinap, Argentina  B-mode  Prosound Alpha-10; Aloka, Japan; manually, caliper  Philips Medical Systems’ HD11 platform; B-mode  B-mode  Pro-sound alpha6 Hitachi Aloka, Switzerland; B-mode  GE Medical Systems, Wauwatosa, WI; B-mode  ATL 5000 HD, manually  Sonos 5500 HP, HP Philips, Netherlands; manually  Combined 2D-mode and color Doppler  Logiq E9 GE Medical Systems, WI, USA; S.A.  Syngo US Workplace Siemens; manually /S.A.  MicroMaxx Sonosite Inc., Bothell, WA; caliper  GE Medical Systems, Horten, Norway; B-mode  Not specified  Acuson Sequoia 512 Siemens USA; S.A.  Logiq Book XP, GE Germany; S.A. Sigma Scan Pro  ATL 5000 HDI device; manually  HR US Acuson Sequoia, Philips TL 5000; B-mode  HDI 5000 ATL, Bothell, WA; not specified | A, R  A, R  B, R&L  B, L  C, R&L  B, R&L  B, N/A  G, R&L  C, R&L  A, R&L  C, R&L  A, N/A  A, N/A  C, L  C, R&L  A, R&L  D, R&L  A/B, L  E, R  C, R&L  C, R&L  F, R&L |

**References:**

- Farello G, Antenucci A, Stagi S, et al. Metabolically healthy and metabolically unhealthy obese children both have increased carotid intima-media thickness: A case control study. BMC Cardiovasc Disord 2018;18(1):1-6.
- Garcia-Espinosa V, Bia D, Castro J, et al. Peripheral and Central Aortic Pressure, Wave-Derived Reflection Parameters, Local and Regional Arterial Stiffness and Structural Parameters in Children and Adolescents: Impact of Body Mass Index Variations. High Blood Press Cardiovasc Prev 2018;25(3):267-80.
- Gooty VD, Sinaiko AR, Ryder JR, et al. Association Between Carotid Intima Media Thickness, Age, and Cardiovascular Risk Factors in Children and Adolescents. Metab Syndr Relat Disord 2018;16(3):122-6.
- Calabrò MP, Carerj S, Russo MS, et al. Carotid artery intima-media thickness and stiffness index β changes in normal children: role of age, height and sex. J Cardiovasc Med 2017;18(1):19-27.
- Baroncini LAV, Sylvestre LC, Pecoits-Filho RF. Assessment of intima–media thickness in healthy children aged 1 to 15years old. BBA Clin 2015;3:S1-S2.
- Curcio S, García-Espinosa V, Arana M, et al. Growing-Related Changes in Arterial Properties of Healthy Children, Adolescents, and Young Adults Nonexposed to Cardiovascular Risk Factors: Analysis of Gender-Related Differences. Int J Hypertens 2016.
- Dalla Pozza R, Pirzer R, Beyerlein A, et al. Beyond intima-media-thickness: Analysis of the carotid intima-media-roughness in a paediatric population. Atherosclerosis 2016;251:164-9.
- Gao Z, Khoury PR, McCoy CE, et al. Adiposity has no direct effect on carotid intima-media thickness in adolescents and young adults: Use of structural equation modeling to elucidate indirect & direct pathways. Atherosclerosis 2016;246:29-35.
- Śladowska-Kozłowska J, Litwin M, Niemirska A, et al. Associations of the eNOS G894T gene polymorphism with target organ damage in children with newly diagnosed primary hypertension. Pediatr Nephrol 2015;30(12):2189-97.
- Alp H, Eklioğlu BS, Atabek ME, et al. Evaluation of epicardial adipose tissue, carotid intima-media thickness and ventricular functions in obese children and adolescents. J Pediatr Endocrinol Metab 2014;27(9-10):827-35.
- Atabek ME, Akyürek N, Eklioglu BS, et al. Impaired systolic blood dipping and nocturnal hypertension: An independent predictor of carotid intima-media thickness in type 1 diabetic patients. J Diabetes Complications 2014;28(1):51-5.
- Koçyiğit A, Doğan M, Yilmaz İ, et al. Relation of age and sex with carotid intima media thickness in healthy children. Turk J Med Sci 2014;44(3):422-6.
- Doyon A, Kracht D, Bayazit AK, et al. Carotid Artery Intima-Media Thickness and Distensibility in Children and Adolescents. Hypertension 2013;62(3):550-6.
- Kollias A, Psilopatis I, Karagiaouri E, et al. Adiposity, blood pressure, and carotid intima-media thickness in greek adolescents. Obesity 2013;21(5):1013-7.
- Sarkola T, Slorach C, Hui W, et al. Transcutaneous very-high resolution ultrasound for the quantification of carotid arterial intima-media thickness in children - feasibility and comparison with conventional high resolution vascular ultrasound imaging. Atherosclerosis 2012;224(1):102-7.
- Pacifico L, Anania C, Osborn JF, et al. Low 25(OH)D3 levels are associated with total adiposity, metabolic syndrome, and hypertension in Caucasian children and adolescents. Eur J Endocrinol 2011;165(4):603-11.
- Mittelman SD, Gilsanz P, Mo AO, et al. Adiposity Predicts Carotid Intima-Media Thickness in Healthy Children and Adolescents. J Pediatr 2010;156(4): 292-7.
- Böhm B, Hartmann K, Buck M, et al. Sex differences of carotid intima-media thickness in healthy children and adolescents. Atherosclerosis 2009;206(2):458-63.
- Litwin M, Niemirska A. Intima - Media thickness measurements in children with cardiovascular risk factors. Pediatr Nephrol 2009;24(4):707-19.
- Jourdan C, Schenk JP, Tröger J, et al. Normative values for intima-media thickness and distensibility of large arteries in healthy adolescents. J Hypertens 2005;23(9):1707-15.
- Pilz S, Horejsi R, Möller R, et al. Early atherosclerosis in obese juveniles is associated with low serum levels of adiponectin. J Clin Endocrinol Metab 2005;90(8):4792-6.

| **B: Under 10 years** | | | |
| --- | --- | --- | --- |
| **Research** | **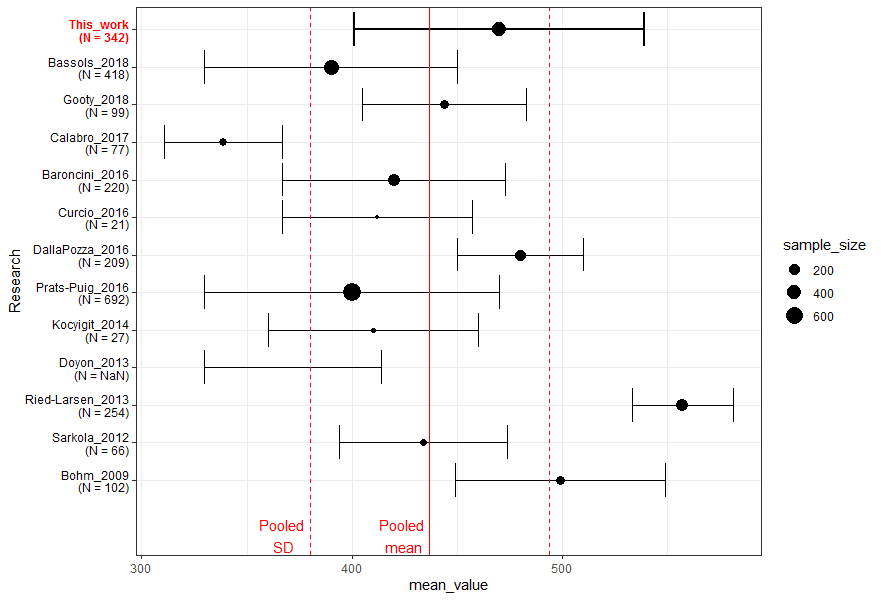Pooled mean cIMT (µm) and SD** | **Reported device and method of cIMT measurement** | **Site of measurement on the CCA** |
| 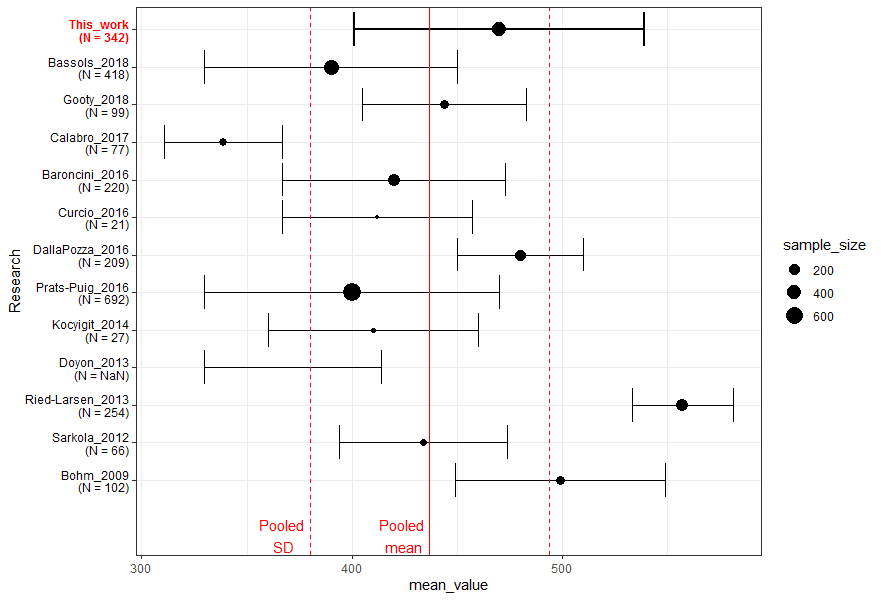 | | RF-QIMT, Esaote, Italy; S.A.  MyLabTM25, Esaote, Firenze, Italy  B-mode  Prosound Alpha-10; Aloka, Japan; manually, caliper  Philips Medical Systems’ HD11 platform; B-mode  B-mode  Pro-sound alpha6 Hitachi Aloka, Switzerland; B-mode  MyLabTM25, Esaote, Firenze, Italy  Logiq E9 GE Medical Systems, WI, USA; S.A.  Syngo US Workplace Siemens; manually /S.A.  Model Logic e GE Medical; Vascular ResearchTools LLC  GE Medical Systems, Horten, Norway; B-mode  Logiq Book XP, GE Germany; S.A. Sigma Scan Pro | A, R  B, R  B, L  C R&L  B, R&L  B, N/A  G, R&L  B, R  A, N/A  C, L  G, R&L  A, R&L  E, R |

**References:**

- Bassols J, Martínez-Calcerrada JM, Prats-Puig A, et al. Perirenal fat is related to carotid intima-media thickness in children. Int J Obes 2018;42(4):641-7.
- Gooty VD, Sinaiko AR, Ryder JR, et al. Association Between Carotid Intima Media Thickness, Age, and Cardiovascular Risk Factors in Children and Adolescents. Metab Syndr Relat Disord 2018;16(3):122-6.
- Calabrò MP, Carerj S, Russo MS, et al. Carotid artery intima-media thickness and stiffness index β changes in normal children: role of age, height and sex. J Cardiovasc Med 2017;18(1):19-27.
- Baroncini LAV, Sylvestre LC, Pecoits-Filho RF. Assessment of intima–media thickness in healthy children aged 1 to 15years old. BBA Clin 2015;3:S1-S2.
- Curcio S, García-Espinosa V, Arana M, et al. Growing-Related Changes in Arterial Properties of Healthy Children, Adolescents, and Young Adults Nonexposed to Cardiovascular Risk Factors: Analysis of Gender-Related Differences. Int J Hypertens 2016.
- Dalla Pozza R, Pirzer R, Beyerlein A, et al. Beyond intima-media-thickness: Analysis of the carotid intima-media-roughness in a paediatric population. Atherosclerosis 2016;251:164-9.
- Prats-Puig A, Moreno M, Carreras-Badosa G, et al. Serum Ferritin Relates to Carotid Intima-Media Thickness in Offspring of Fathers with Higher Serum Ferritin Levels. Arterioscler Thromb Vasc Biol. 2016;36(1):174-180.
- Koçyiğit A, Doğan M, Yilmaz İ, et al. Relation of age and sex with carotid intima media thickness in healthy children. Turk J Med Sci 2014;44(3):422-6.
- Doyon A, Kracht D, Bayazit AK, et al. Carotid Artery Intima-Media Thickness and Distensibility in Children and Adolescents. Hypertension 2013;62(3):550-6.
- Ried-Larsen M, Grøntved A, Møller NC, et. al. Associations between objectively measured physical activity intensity in childhood and measures of subclinical cardiovascular disease in adolescence: Prospective observations from the European Youth Heart Study. Br J Sports Med. 2014;48(20):1502-7.
- Sarkola T, Slorach C, Hui W, et al. Transcutaneous very-high resolution ultrasound for the quantification of carotid arterial intima-media thickness in children - feasibility and comparison with conventional high resolution vascular ultrasound imaging. Atherosclerosis 2012;224(1):102-7.
- Böhm B, Hartmann K, Buck M, et al. Sex differences of carotid intima-media thickness in healthy children and adolescents. Atherosclerosis 2009;206(2):458-63.

| **C: Age group 10-14 years** | | | |
| --- | --- | --- | --- |
| **Research** | **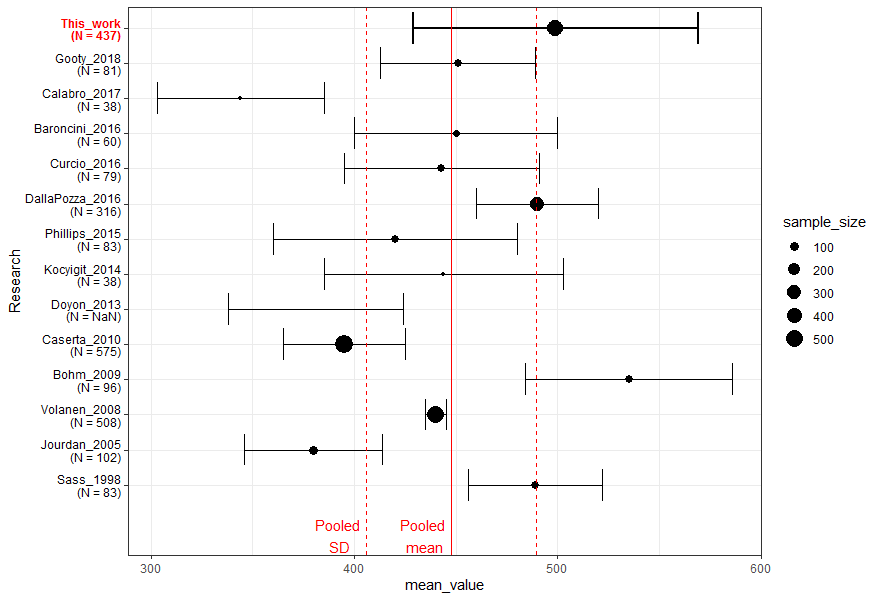Pooled mean cIMT (µm) and SD** | **Reported device and method of cIMT measurement** | **Site of measurement on the CCA** |
| 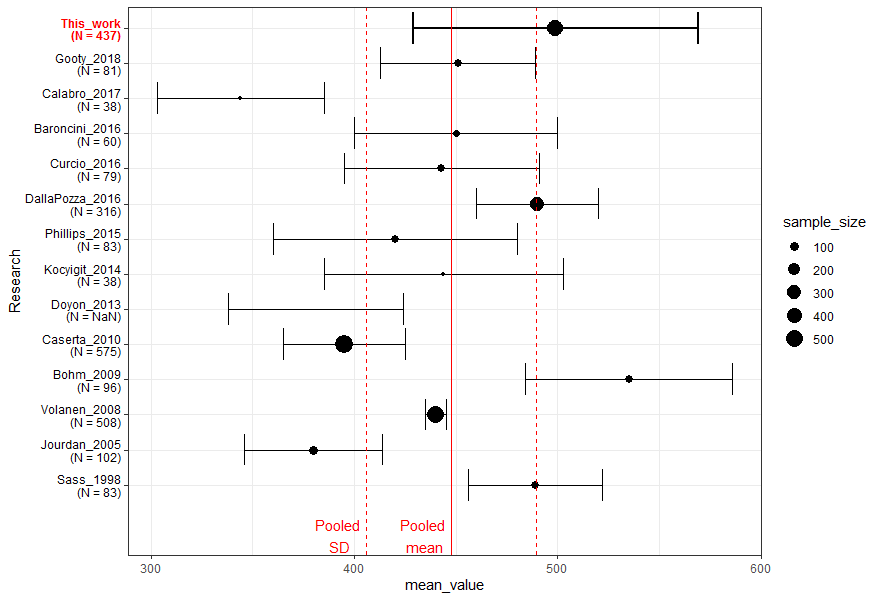 | | RF-QIMT, Esaote, Italy; S.A.  B-mode  Prosound Alpha-10; Aloka, Japan; manually, caliper  Philips Medical Systems’ HD11 platform; B-mode  B-mode  Pro-sound alpha6 Hitachi Aloka, Switzerland, B-mode  Vivid i,General Electric Medical Systems, Ne  Logiq E9 GE Medical Systems, WI, USA; S.A.  Syngo US Workplace Siemens; manually /S.A.  Mylab 25 Esaote, Genova, Italy; S.A.  Logiq Book XP, GE Germany; S.A. Sigma Scan Pro  Acuson Sequoia 512, CA  HR US Acuson Sequoia, Philips TL 5000; B-mode  HITASHI EUB 565, Iotec, France; B-mode | A, R  B, L  C R&L  B, R&L  B, N/A  G, R&L  G, R  A, N/A  C, L  H, R&L  E, R  G, R&L, dif. ang.  C, R&L  I, R&L |

**References:**

- Gooty VD, Sinaiko AR, Ryder JR, et al. Association Between Carotid Intima Media Thickness, Age, and Cardiovascular Risk Factors in Children and Adolescents. Metab Syndr Relat Disord 2018;16(3):122-6.
- Calabrò MP, Carerj S, Russo MS, et al. Carotid artery intima-media thickness and stiffness index β changes in normal children: role of age, height and sex. J Cardiovasc Med 2017;18(1):19-27.
- Baroncini LAV, Sylvestre LC, Pecoits-Filho RF. Assessment of intima–media thickness in healthy children aged 1 to 15years old. BBA Clin 2015;3:S1-S2.
- Curcio S, García-Espinosa V, Arana M, et al. Growing-Related Changes in Arterial Properties of Healthy Children, Adolescents, and Young Adults Nonexposed to Cardiovascular Risk Factors: Analysis of Gender-Related Differences. Int J Hypertens 2016.
- Dalla Pozza R, Pirzer R, Beyerlein A, et al. Beyond intima-media-thickness: Analysis of the carotid intima-media-roughness in a paediatric population. Atherosclerosis 2016;251:164-9.
- Phillips AA, Chirico D, Coverdale NS, et al. The association between arterial properties and blood pressure in children. Appl Physiol Nutr Metab 2015;40(1):72-8.
- Koçyiğit A, Doğan M, Yilmaz İ, et al. Relation of age and sex with carotid intima media thickness in healthy children. Turk J Med Sci 2014;44(3):422-6.
- Doyon A, Kracht D, Bayazit AK, et al. Carotid Artery Intima-Media Thickness and Distensibility in Children and Adolescents. Hypertension 2013;62(3):550-6.
- Caserta CA, Pendino GM AS. Body mass index, cardiovascular risk factors, and carotid intima-media thickness in a pediatric population in southern Italy. J Pediatr Gastro- enterol Nutr. 2010;51:216–20.
- Böhm B, Hartmann K, Buck M, et al. Sex differences of carotid intima-media thickness in healthy children and adolescents. Atherosclerosis 2009;206(2):458-63.
- Volanen I, Kallio K, Saarinen M, et al. Arterial intima-media thickness in 13-year-old adolescents and previous antichlamydial antimicrobial use: a retrospective follow-up study. Pediatrics 2008;122(3):e675-81.
- Jourdan C, Schenk JP, Tröger J, et al. Normative values for intima-media thickness and distensibility of large arteries in healthy adolescents. J Hypertens 2005;23(9):1707-15.
- Sass C, Herbeth B, Chapet O, et al. Intima media thickness and diameter of carotid and femoral arteries in children, adolescents and adults from the Stanislas cohort. Hypertension 1998;16(11):1593–1602.

| **D: Age group 14-18 years** | | | |
| --- | --- | --- | --- |
| **Research** | **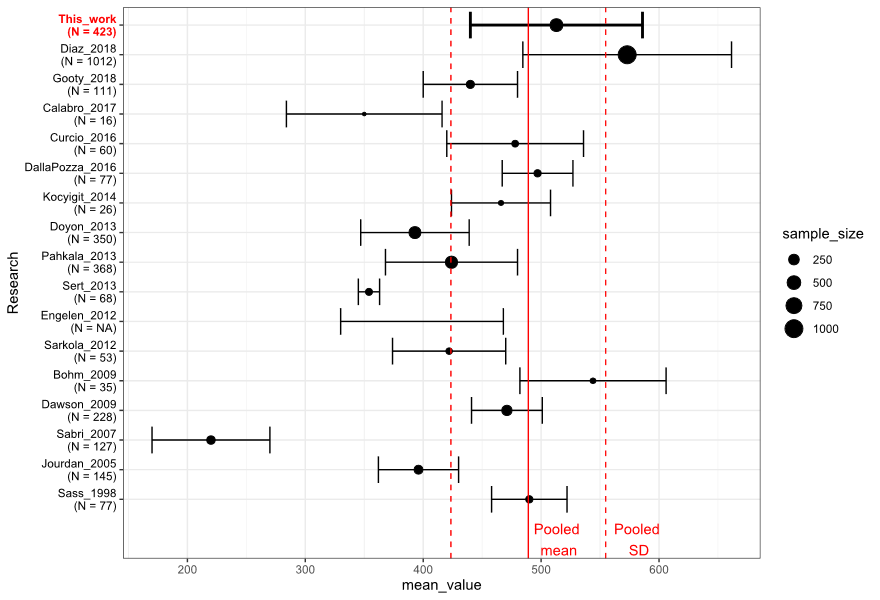Pooled mean cIMT (µm) and SD** | **Reported device and method of cIMT measurement** | **Site of measurement on the CCA** |
| 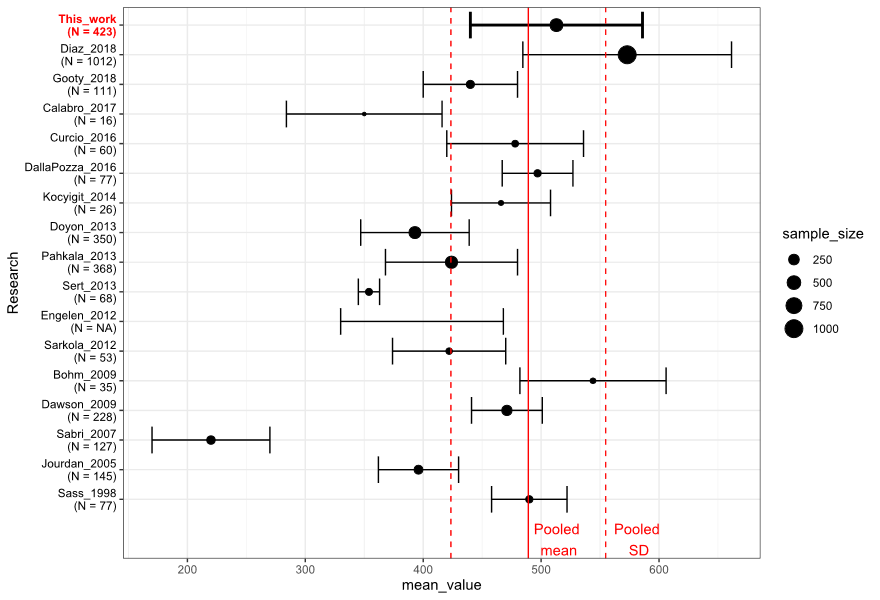 | | RF-QIMT, Esaote, Italy; S.A.  Esaote MyLab 40; S.A.  End diastole; B-mode  Prosound Alpha-10; Aloka, Japan; manually, caliper  B-mode  Pro-sound alpha6 Hitachi Aloka, Switzerland; B-mode  Logiq E9 GE Medical Systems, WI, USA; S.A.  Syngo US Workplace Siemens; manually /S.A.  Acuson Sequoia 512, CA  Logiq 7 GE Healthcare, Wisconsin; N/A  Multiple  GE Medical Systems, Horten, Norway; B-mode  Logiq Book XP, GE Germany; S.A. Sigma Scan Pro  B-mode  B-mode  HR US Acuson Sequoia, Philips TL 5000; B-mode  HITASHI EUB 565, Iotec, France; B-mode | A, R  B, R&L  B, L  C R&L  B, N/A  G, R&L  A, N/A  C, L  G, R&L  A, N/A  Multiple  A, R&L  E, R  Multiple  B, R&L  C, R&L  I, R&L |

**References:**

- Diaz A, Bia D, Zócalo Y, et al. Carotid Intima Media Thickness Reference Intervals for a Healthy Argentinean Population Aged 11–81 Years. Int J Hypertens 2018;2018:1-13.
- Gooty VD, Sinaiko AR, Ryder JR, et al. Association Between Carotid Intima Media Thickness, Age, and Cardiovascular Risk Factors in Children and Adolescents. Metab Syndr Relat Disord 2018;16(3):122-6.
- Calabrò MP, Carerj S, Russo MS, et al. Carotid artery intima-media thickness and stiffness index β changes in normal children: role of age, height and sex. J Cardiovasc Med 2017;18(1):19-27.
- Curcio S, García-Espinosa V, Arana M, et al. Growing-Related Changes in Arterial Properties of Healthy Children, Adolescents, and Young Adults Nonexposed to Cardiovascular Risk Factors: Analysis of Gender-Related Differences. Int J Hypertens 2016.
- Dalla Pozza R, Pirzer R, Beyerlein A, et al. Beyond intima-media-thickness: Analysis of the carotid intima-media-roughness in a paediatric population. Atherosclerosis 2016;251:164-9.
- Koçyiğit A, Doğan M, Yilmaz İ, et al. Relation of age and sex with carotid intima media thickness in healthy children. Turk J Med Sci 2014;44(3):422-6.
- Doyon A, Kracht D, Bayazit AK, et al. Response to Intima–Media Thickness in Children—Need for More Parameters. Hypertension 2014;63(5):121-3.
- Pahkala K, Laitinen TT, Heinonen OJ, et al. Association of fitness with vascular intima-media thickness and elasticity in adolescence. Pediatrics 2013;132(1):e77-84.
- Sert A, Aypar E, Pirgon O, et al. Left ventricular function by echocardiography, tissue Doppler imaging, and carotid intima-media thickness in obese adolescents with nonalcoholic fatty liver disease. Am J Cardiol 2013;112(3):436-43.
- Engelen L, Ferreira I, Stehouwer CD, et al. Reference intervals for common carotid intima-medi thickness measured with echotracking: Relation with risk factors. Eur Heart J 2013;34(30):2368-80.
- Sarkola T, Slorach C, Hui W, et al. Transcutaneous very-high resolution ultrasound for the quantification of carotid arterial intima-media thickness in children - feasibility and comparison with conventional high resolution vascular ultrasound imaging. Atherosclerosis 2012;224(1):102-7.
- Böhm B, Hartmann K, Buck M, et al. Sex differences of carotid intima-media thickness in healthy children and adolescents. Atherosclerosis 2009;206(2):458-63.
- Dawson, JD, Sonka M, Blecha, MB, et al. Risk Factors Associated With Aortic and Carotid Intima-Media Thickness in Adolescents and Young Adults. J Am Coll Cardiol 2009;53(24):2273–9.
- Sabri MR. The thickness of the intimal and medial layers of the carotid arteries, and the index of left ventricular mass, in children of patients with premature coronary arterial disease. Cardiol Young 2007;17(6):609-16.
- Jourdan C, Schenk JP, Tröger J, et al. Normative values for intima-media thickness and distensibility of large arteries in healthy adolescents. J Hypertens 2005;23(9):1707-15.
- Sass C, Herbeth B, Chapet O, et al. Intima media thickness and diameter of carotid and femoral arteries in children, adolescents and adults from the Stanislas cohort. Hypertension 1998;16(11):1593–1602.
